# INJECTABLE LONG-ACTING IVACAFTOR-LOADED POLY (LACTIDE-CO-GLYCOLIDE) MICROPARTICLE FORMULATIONS FOR THE TREATMENT OF CYSTIC FIBROSIS: IN VITRO CHARACTERIZATION AND IN VIVO PHARMACOKINETICS IN MICE

**DOI:** 10.1016/j.ijpharm.2023.123693

**Published:** 2023-12-09

**Authors:** David S. Nakhla, Aml I. Mekkawy, Youssef W. Naguib, Aaron D. Silva, Dylan Gao, Jeong Ah Kim, Suhaila O. Alhaj-Suliman, Timothy M. Acri, Krishna Kumar Patel, Sarah Ernst, David A. Stoltz, Michael J. Welsh, Aliasger K. Salem

**Affiliations:** 1 Department of Pharmaceutical Sciences and Experimental Therapeutics, College of Pharmacy, University of Iowa, Iowa City, IA 52242, USA; 2 Department of Pharmaceutics and Clinical Pharmacy, Faculty of Pharmacy, Sohag University, Sohag, Sohag 82524, Egypt; 3 Holden Comprehensive Cancer Center, University of Iowa, Iowa City, IA 52242, USA; 4 Howard Hughes Medical Institute, Roy J. and Lucille A. Carver College of Medicine University of Iowa, Iowa City, Iowa 52242; 5 Department of Internal Medicine, Roy J. and Lucille A. Carver College of Medicine University of Iowa, Iowa City, Iowa 52242; 6 Departments of Molecular Physiology and Biophysics, Roy J. and Lucille A. Carver College of Medicine University of Iowa, Iowa City, Iowa 52242

**Keywords:** ivacaftor, cystic fibrosis, long-acting injectables (LAI), PLGA microparticles, pharmacokinetics

## Abstract

Optimizing a sustained-release drug delivery system for the treatment of cystic fibrosis is crucial for decreasing the dosing frequency and improving patients’ compliance with the treatment regimen. In the current work, we developed an injectable PLGA microparticle formulation loaded with ivacaftor, a CFTR potentiator that increases the open probability of the CFTR anion channel, using a single emulsion solvent evaporation technique. We aimed to study the effect of different parameters on the characteristics of the prepared formulations to select an optimized microparticle formulation to be used in the *in vivo* pharmacokinetic study in mice. First, ivacaftor-loaded microparticles were prepared while varying the formulation parameters to study their effect on the formulations’ size, morphology, drug loading, encapsulation efficiency, and *in vitro* release profiles. All the prepared microparticles showed smooth spherical surfaces with internal diameters of 1.91– 6.93 μm, drug loading (DL) of 3.91 – 10.3%, percent encapsulation efficiencies (%EE) of 26.6 – 100%, and an overall slow cumulative release profile. We selected one formulation that showed the best combined %DL and %EE values (8.25, and 90.7%, respectively), with an average particle size of 6.83 μm, and a slow bi-phasic in vitro release profile (up to 6 weeks) to study its in vivo pharmacokinetics in comparison to solubilized ivacaftor following their subcutaneous (SC) and intravenous (IV) administration to mice, respectively. The injected microparticle formulation showed steady plasma levels of ivacaftor over a period of 28 days, and a 6-fold increase in AUC _0 – t_ (71.6 μg/mL*h) compared to the intravenously injected soluble ivacaftor (12.3 μg/mL*h). Our results suggest that this novel ivacaftor-loaded microparticle formulation could potentially eliminate the need for the frequent daily administration of ivacaftor by people with cystic fibrosis which could improve their compliance and ensure successful treatment outcomes.

## Graphical Abstract

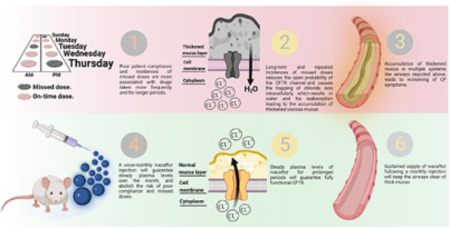

## 1. Introduction

**Scheme 1:**
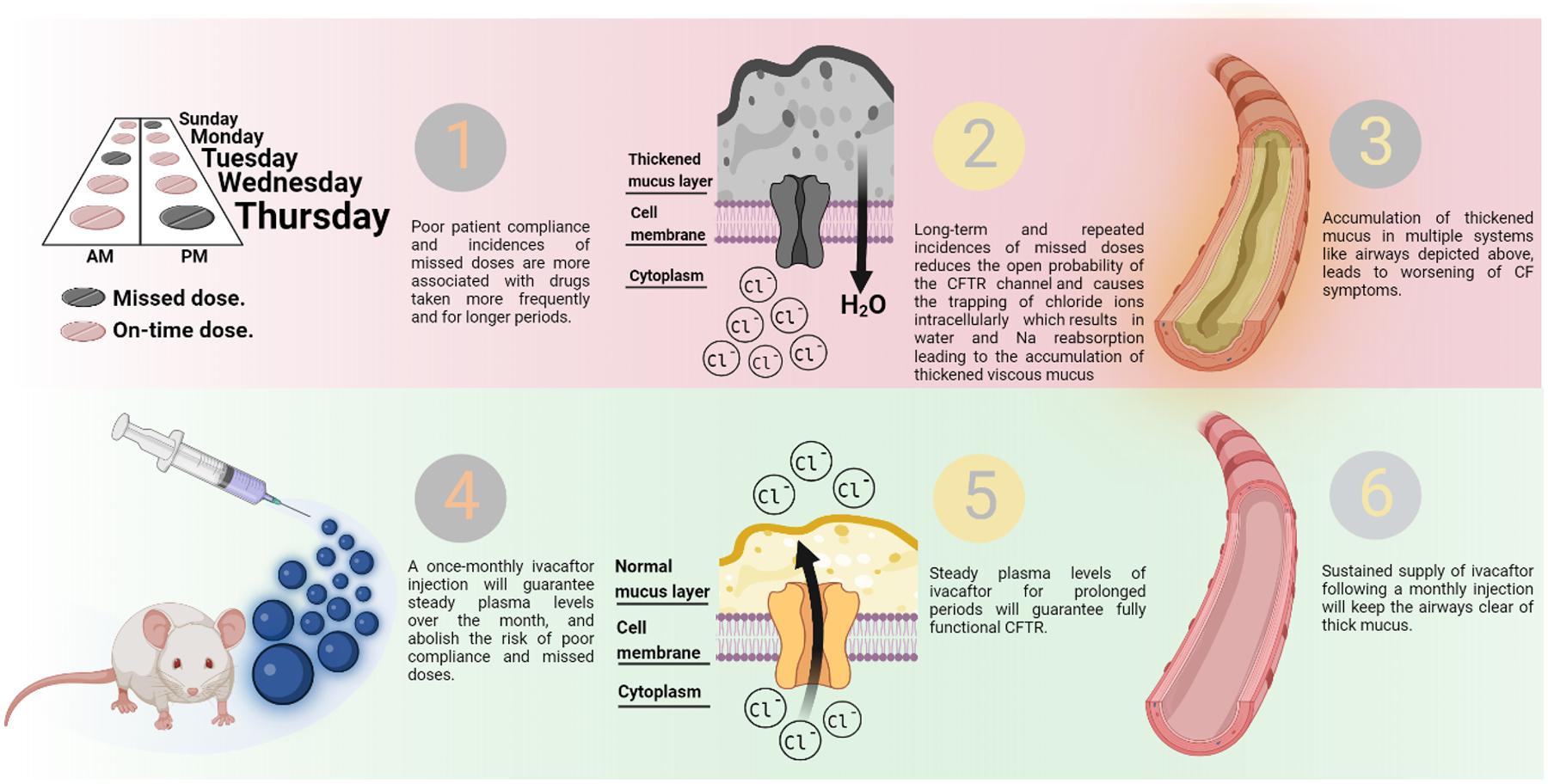
A schematic diagram showing the pathogenesis of cystic fibrosis and the benefits of using a once per month injectable ivacaftor-loaded microparticles on the prognosis of the disease.

Cystic fibrosis (CF) is an autosomal recessive disease caused by a mutation in the gene encoding the cystic fibrosis transmembrane conductance regulator (CFTR) anion channel, which is responsible for maintaining water and ion balance intra- and extracellularly (Ikpa, Bijvelds, and de Jonge 2014). This serious life-shortening condition affects 30,000 people in the United States of America and 70,000 worldwide. Loss of CFTR function prevents chloride and bicarbonate flow across epithelial cells resulting in apical mucus accumulation and disrupted function of multiple organs including pulmonary, gastrointestinal, hepatic, and reproductive systems (Scheme 1) (Condren and Bradshaw 2013). Currently, there are more than 1900 disease-causing *CFTR-*mutations. These mutations are grouped into five classes based on the type of mutation and its resulting phenotype. Class I mutation involves truncated protein translation, while class II mutations cause misfolded CFTR. These two defects result in the inability of the CFTR protein to translocate to the cell surface. The third and fourth classes’ defects result in a fully translated CFTR able to translocate to the cell surface, but it exhibits gating dysfunction or decreased pore conductivity. Class V defects cause a reduction in CFTR protein expression at the cell surface (Condren and Bradshaw 2013).

Ivacaftor [VX-770, *N*-(2,4-Di-tert-butyl-5-hydroxyphenyl)-4-oxo-1,4-dihydroquinoline-3-carboxamide] is a selective small molecule CFTR modulator that targets the 3^rd^ most common genetic mutation in the CFTR gene (G551D – Class III – gating mutation, occurring in 5–6% of people with CF) (Clancy et al. 2014; Fohner et al. 2017). It restores the defective CFTR function by increasing the open probability of CFTR channels at the cell surface (CFTR ‘potentiator’). It was the first CFTR modulator to be approved in 2012 after being developed by Vertex pharmaceuticals, formulated into amorphous solid dispersion tablets, and sold under the trade name Kalydeco^®^ (Deeks 2016; Fohner et al. 2017). Kalydeco^®^ tablets are orally administered at a dose of 150 mg twice daily owing to the short half-life of ivacaftor (9–12 h) (Deeks 2016), and they are recommended to be taken with fatty meals to improve ivacaftor’s stomach absorption and bioavailability due to the drug’s low water solubility (<0.05 μg/mL) and high lipophilicity (logP = 3.3). Furthermore, it has been reported that treatment adherence rates to Kalydeco^®^ by people with CF is surprisingly suboptimal (about 61%), which negatively influences the overall health and quality of life of these individuals (Siracusa et al. 2015). Treatment adherence of children with cystic fibrosis is even less (<50%), which could be related to the chronic nature of the disease, and the complexity of the dosing regimens (Modi et al. 2006)(Conway et al. 1996). In addition, ivacaftor is a substrate for cytochrome P-450 (CYP3A) enzymes which makes it prone to 1- first-pass metabolism which decreases its oral bioavailability, and 2- food-drug interactions if orally administered along with CYP-inducers or inhibitors (e.g., Seville oranges and grapefruit) resulting in sub-therapeutic or toxic drug concentrations, respectively (Condren and Bradshaw 2013; Kapoor, Koolwal, and Singh 2014). Developing an extended-release injectable formulation loaded with ivacaftor could potentially be a better alternative than the oral administration of Kalydeco^®^ tablets. This is because the long-acting injectable ivacaftor formulation will potentially 1- improve ivacaftor’s bioavailability by avoiding the first-pass metabolism and providing sustained plasma levels of the drug over a long period of time, and 2- eliminate the need for frequent daily administrations of the drug and thereby improve patients’ convenience and compliance with the treatment regimen as well as ensure positive treatment outcomes.

In this research, we set out to encapsulate ivacaftor into an injectable, biodegradable, and long-acting poly (D,L-lactide-*co*-glycolide) (PLGA) microparticle-based formulation. The synthetic PLGA copolymer has been widely used in injectable long-acting formulations for more than 3 decades, either in microparticles, in situ forming implants, or solid implants. It is mainly used to encapsulate small molecules (hydrophilic and hydrophobic), as well as macromolecules like; proteins, peptides, and antigens (as adjuvants in cancer vaccines and immunotherapy) (Gross et al. 2020; Joshi, Geary, and Salem 2013; Naguib, Givens, et al. 2020; Naguib, Yu, et al. 2020; Park et al. 2020a). One of the main advantages of using PLGA in developing long-acting drug delivery systems is the ability to tailor the release profile of the encapsulated drug by controlling the polymer chemistry including the polymer’s lactide:glycolide ratio (L:G ratio), molecular weight, and end-group functionalization (Lagreca et al. 2020). In addition, long-acting formulations avoid toxic or subtherapeutic effects that can result from repeated administration of drugs due to the sharp fluctuations in plasma drug concentrations, by providing steady plasma levels over time (Naguib, Yu, et al. 2020; Park et al. 2019, 2020a). Porsio and Zhu et al, have recently encapsulated ivacaftor into inhalable nano-into-micro dry powders, nanocomposite microparticles as well as mucus penetrating nanoparticles for the goal of enhancing its pulmonary delivery(Porsio et al. 2018, 2020; Zhu et al. 2020). To the best of our knowledge, this is the first study that reports the development of an injectable PLGA-based long-acting ivacaftor-loaded microparticle formulation. To develop an optimum microparticle formulation, we studied the effect of various formulation parameters on the characteristics of the prepared microparticles’, including particle size, drug loading (DL), percent encapsulation efficiency (%EE), and *in vitro* drug release profile. The selected formulation was further subjected to *in vivo* pharmacokinetic study after subcutaneous (SC) injection in mice. The pharmacokinetic parameters of the SC-injected ivacaftor-loaded microparticles were compared with those of the intravenously injected soluble ivacaftor (solubilized in 10% v/v polysorbate 80 in sterile phosphate-buffered saline (PBS), pH 7.4).

## 2. Materials and Methods

### 2.1 Materials

Ivacaftor was purchased from Matrix Scientific (Columbia, SC). Poly lactide-co-glycolide (PLGA, Resomer^®^ RG 503 H, MW= 24,000 – 38,000, viscosity= 0.32–0.44 dL/g in chloroform and Resomer^®^ RG 502 H, MW = 7,000 – 17,000, viscosity = 0.16–0.24 dL/g in chloroform) were purchased from Evonik (Parsippany, NJ). Polyvinyl alcohol (PVA, Mowiol 8–88, M.Wt 67,000), screw-capped dialysis tube (Spectra/PorTM Float-A-LyzerTM G2, MWCO 50 kDa), and phosphate-buffered saline (PBS) tablets were purchased from Sigma Aldrich (St. Louis, MO). Tween^®^ 80, methylene chloride (dichloromethane, DCM), and acetonitrile were obtained from Fisher Chemicals (Waltham, MA). Balb C Mouse plasma (Na Heparin) was obtained from Innovative research, Inc., (Novi, MI). All other chemicals and reagents used were of analytical or HPLC grade.

### 2.2 Methods

#### 2.2.1 Preparation and characterization of ivacaftor-loaded PLGA microparticles

Ivacaftor-loaded PLGA microparticles were prepared using an oil-in-water single emulsion solvent evaporation technique (Supplemental Figure 1) as previously described (Khaled et al. 2010; Naguib et al. 2021), using different formulation parameters as described in Table 1. Briefly, 5 or 10 mg ivacaftor and 50 mg of PLGA polymer were dissolved in 1.5 mL of DCM (the organic phase). The organic solution was added to 30 mL of aqueous PVA solution (1 or 1.5% w/v) and the mixture was immediately emulsified for 1 min at different homogenizing speeds (6500 or 13500 rpm) at room temperature using an overhead homogenizer (Ultra-turrax T25 basic, Ika Works, Inc., Wilmington, NC). The emulsion was kept stirring at a speed of 300 rpm using a magnetic digital stirrer for 2 h at 25 °C to evaporate the organic solvent. Microparticles were then collected by centrifugation of the suspension at 1000 x g for 5 min (Eppendorf centrifuge 5864 R, Eppendorf North America, Hauppauge, NY), washed three times with 30 mL Nanopure water (Barnstead Thermolyne Nanopure water purification system, Thermo Fisher, Waltham, MA), and freeze-dried overnight till further in vitro studies (Labconco Free zone 4.5L–105 °C, Labconco, Kansas City, MO).

##### Evaluation of particle size and surface morphology

The size, shape, and surface morphology of the prepared PLGA microparticle formulations were investigated using a Hitachi S-4800 scanning electron microscope (SEM, Hitachi High Technologies, Ontario, Canada) (Ahmed et al. 2020; Klose et al. 2006). The lyophilized microparticles were spread onto a carbon double-adhesive tape that is mounted on an aluminum stub, and the surface was coated with gold and palladium using an argon beam K550 sputter coater (Emitech Ltd., Kent, U.K). SEM photomicrographs of the microparticles were captured at 1.5 kV accelerating voltage. A minimum of 100 particles in the captured SEM images were analyzed to obtain the average particle size and standard deviation (SD) using ImageJ software (NIH, Bethesda, MA). The obtained data were plotted in histograms using GraphPad Prism software (GraphPad, San Diego, CA). Particle size distribution was accurately estimated by further analysis of the particle size data using a Microsoft Excel^®^ built-in function to obtain D10, D50, and D90 values which represent the sizes below which 10, 50, and 90% of the microparticles fell. These values were used to calculate the span value which gives an estimate of the dispersibility and uniformity of the microparticles.

The span value was calculated using the following equation:

$$\text{Span}=\frac{D90-D10}{D50}$$

##### Estimation of the drug loading capacity, encapsulation efficiency, and percent yield of Ivacaftor-loaded microparticles

One mg of ivacaftor microparticle formulations was accurately weighed and dissolved in 1 mL acetonitrile while sonicating the solution for 10 min. Then 100 μL of this solution was transferred to 900 μL (500 μL acetonitrile + 400 μL Nanopure water) to make a 10-fold dilution. The solution was vortexed, centrifuged (14,000 x g for 5 min), and analyzed using HPLC as described below. DL, percent encapsulation efficiency (%EE), and percent yield of the microparticles were determined using the following equations:

$$\text{Drug Loading}\left(\frac{ug}{mg}\right)=\frac{\text{Ivacaftor Concentration}\left(\frac{ug}{mL}\right)\times \text{volume}(mL)}{\text{Particles weight}(\text{mg})}$$

$$\text{Yield}(\%)=\frac{\text{Weight of Lyophilized particles}(\text{mg})}{\text{Weight of starting materials}(\text{mg})}\times 100$$

$$\text{Encapsulation ef ficiency}(\%)=\frac{\text{Drug loading}\left(\frac{ug}{mg}\right)\times \text{Total weight or YIELD of particles}(\text{mg})}{\text{Initial amount of drug added}(\text{ug})}\times 100$$

$$\text{Drug loading}(\%)=\frac{\text{Drug amount}(\text{mg})}{\text{Total weight of particles}(\text{mg})}\times 100$$

##### Differential scanning calorimetry (DSC)

Samples of 3 – 5 mg of ivacaftor microparticles (F1), PLGA (Resomer^®^ 503H), their physical mixture, and pure ivacaftor were accurately weighed, transferred to a Tzero aluminum pan, covered with a lid, and compressed. Thermograms were then obtained using a differential scanning calorimeter (DSC) (TA DSC instrument model Q20, New Castle, DE) coupled with a refrigerated cooling system (RCS90). Compressed empty pan and lid were used as a reference. All samples were heated in the scanning range of 25 °C – 325 °C at a 10 °C/min heating rate. The pressure of the pure dry nitrogen purge gas was set at 20 psi at a flow rate of 40 mL/min.

##### Powder X-ray diffraction (PXRD)

A Siemens D5000 diffractometer was used to obtain the X-ray powder diffraction patterns of ivacaftor microparticles (F1), PLGA (Resomer^®^ RG503H), pure ivacaftor, and PLGA/ivacaftor physical mixture. An X-ray source composed of Cu Kα X-rays with 1.51418 Å was used and the diffraction patterns (diffractograms) of all samples were recorded in the range of 5° – 50° at 2ϴ values with a step size of 0.02° and a dwell time of 0.5 s (Young, Bahl, and Stevens 2019).

#### 2.2.2 In vitro release study

Ivacaftor water’s solubility is 270 μg/mL. An accurately weighed amount of the prepared ivacaftor-loaded PLGA microparticles (equivalent to 270 μg ivacaftor) was suspended in 1 mL of DPBS (1x Dulbecco’s phosphate-buffered saline, Life Science, Waltham, MA). The microparticles suspension was then transferred to a 1 mL screw-capped dialysis tube (Spectra/Por^™^ Float-A-Lyzer^™^ G2 MWCO 50 kDa, Sigma-Aldrich), and the tubes were immersed in a 50 mL tube containing 12 mL of DPBS pH 7.4 + 0.4% v/v Tween^®^ 80 to maintain sink conditions. The tubes were then transferred to an orbital shaker (New Brunswick Scientific, Edison, NJ) set at 300 rpm and 37 °C. At predetermined time points of 0.042, 0.125, 0.25, 1, 2, 3, 7, 10, 14, 17, 21, 24, and 30 days, one mL of the release media from each tube was withdrawn for drug analysis, and the whole remaining release medium was replaced with fresh release medium. Ivacaftor concentration was determined using the HPLC method as described below. Each formulation was prepared as three independent batches, and each batch was independently tested. The data were represented as the average cumulative release (%) ± SD.

#### 2.2.3 In vitro release modeling

The ivacaftor cumulative in vitro drug release data were fitted to 5 release kinetics models (Zero-order, first-order, Korsemeyer-Peppas, Higuchi, and Baker-Londsale) using the DDsolver software (Microsoft Excel^®^ Ad-in) (Zhang, Huo, Zhou, Zou, et al. 2010). All models that were tested are presented in Supplemental Table 1. Since models with a different number of parameters were compared, the model with the lowest Akaike information criterion (AIC) value and highest R-squared (R^2^), was selected as the best-fit model.

#### 2.2.4 In vivo pharmacokinetics

##### Study design

All animal experiments were approved by the University of Iowa Institutional Animal Care and Use Committee (IACUC). Fifty-one male BALB/CJ mice (8 weeks old, weighing 20 – 25 g, Jackson labs, Bar Harbor, ME) were used in the pharmacokinetic studies of soluble ivacaftor and ivacaftor-loaded PLGA microparticles (Formulation 1). Supplemental Figure 2 shows a graphical depiction of the design of the in vivo pharmacokinetics study. Mice have been extensively used in literature for studying the in vivo pharmacokinetics of various drugs (Igarashi et al. 2023; Kim et al. 2023; Lignet et al. 2023; Naguib et al. 2016, 2023). Mice were kept at the University of Iowa animal care facility under controlled temperature (23 ± 2°C) and were exposed to 12 h light and dark cycles. Twenty-one mice were injected intravenously (via tail vein injection) with an ivacaftor solution solubilized in 10% Tween^®^ 80 in sterile 1x DPBS at a concentration of 1 mg/mL (total volume 100 μL for a dose of 5 mg/kg). Three mice were euthanized at each time point (5, 15, 30, 60, 180, 360, 1440 min) by intraperitoneal injection of 100 μL Ketamine/Xylazine (87.5/12.5 mg/kg) cocktail followed by cervical dislocation. Three mice were euthanized at each time point following IACUC guidelines, where only a maximum of 0.2 mL of blood per week is allowed to be collected from each mouse. Our study involved collecting several blood samples within the same day or week to capture the initial distribution and the final elimination phases of the drug. For this reason, we euthanized 3 mice at each time point instead of collecting the blood samples from the same mouse over time. Blood samples were collected immediately via cardiac puncture and transferred to 1.5 mL Eppendorf^®^ tubes containing 10 μL sodium heparin (1000 USP units/mL) to prevent coagulation. Plasma was separated from the supernatant after centrifugation of the blood samples at 14,000 xg for 10 min and frozen at – 80°C until analysis by LC-MS/MS as described below to determine the ivacaftor plasma levels. In contrast, the remaining 30 mice were injected subcutaneously with the microparticles suspension in 1x DPBS at a dose equivalent to 1 mg ivacaftor (50 mg/kg) in 0.3 mL/mouse. At predetermined time points (0.042, 0.167, 1, 7, 10, 14, 21, 24, and 28 days) three mice were sacrificed, and blood samples were collected, processed, and stored as described above, until analysis by LC-MS/MS.

##### Plasma samples preparation

A protein-precipitation technique using acetonitrile was carried out to extract ivacaftor from plasma samples (Naguib et al. 2021). Briefly, plasma samples were thawed and aliquots of 100 μL were transferred to 1.5 mL Eppendorf^®^ tubes and spiked with 10 μL of the internal standard working solution (lumacaftor (VX-809) 2.5 μg/mL for a final concentration of 0.25 μg/mL). Then 1 mL of cold acetonitrile was added, and samples were vortexed for 1 min and transferred to an ice bucket for 10 min to allow plasma proteins precipitation. Samples were then centrifuged at 4,000 xg for 10 min at 4°C, and the supernatant was transferred to glass tubes and evaporated under a light stream of nitrogen. The residue was reconstituted in 100 μL acetonitrile: Water (50:50 v/v), vortexed, centrifuged for 5 min at 14,000 xg, and 20 μL of the supernatant was injected into the LC-MS/MS for quantification.

#### 2.2.5 Analytical methods

##### High-performance liquid chromatography (HPLC)

An HPLC method for the quantification of ivacaftor was developed using an Agilent workstation (Agilent Infinity 1100, Santa Clara, CA) coupled with an Agilent diode array detector (DAD). A reversed-phase (RP) Waters^®^ Symmetry C18 column (5 μm pore size, 4.6 mm ID × 150 mm, Waters, Milford, MA, USA) was used for analysis. The mobile phase consisted of a mixture of Acetonitrile: water (60:40) with 0.1% v/v trifluoroacetic acid in an isocratic elution mode. The flow rate and the injection volume were set at 1 mL/min and 50 μL, respectively, at room temperature. The detection wavelength was set to 309 nm. The stock solution of ivacaftor was prepared in methanol at a concentration of 1 mg/mL. To construct a calibration curve, ivacaftor stock solution was diluted to 6 calibration standards in the range of 0.1 – 50 μg/mL using Acetonitrile: water (50:50 v/v) and injected into the HPLC, data was collected, and a linear regression equation was fit into the calibration standards.

##### Liquid chromatography/tandem mass-spectrometry (LC-MS/MS)

The chromatographic analysis and mass spectrometric detection were carried out using a Waters^®^ Acquity H-class ultra-performance liquid chromatography (UPLC) system coupled with a Waters^®^ XEVO TQ-S cronos triple quadrupole mass spectrometer (Waters Corporation, Milford, MA) operating in positive electrospray (ESI) and multiple reaction monitoring (MRM) mode. Chromatographic separation was performed on an Agilent^®^ RRHD Eclipse Plus C8 column (2.1 ID x 100 mm, 1.8 um pore size, Agilent Technologies, Santa Clara, CA). The mobile phase consisted of (A) water with 0.1 % v/v trifluoroacetic acid and (B) acetonitrile. The initial gradient conditions were 60% (B) for 6 min, gradually increased to 95% over 1 min, and maintained at this concentration for an additional 4 min then switched back to 60% for 6 min to re-equilibrate the column, while the remaining percentage of the mobile phase in the gradient along the run was (A). The total run time was 17 min, and the flow rate was set at 0.2 mL/min with an injection volume of 20 μL/sample.

The mass spectrometric detection of ivacaftor and the internal standard (IS) lumacaftor (VX-809) was conducted in positive ESI via MRM mode. The optimum MRM transitions were *m/z* 393.18 → 172.07 and 453.02 → 131.04 for quantification of ivacaftor and lumacaftor, respectively, and data was acquired between 3.5 and 8 min of the run time. Fragments were induced using a collision energy (CE) of 28 V for ivacaftor and 40 V for lumacaftor. Additional ESI source parameters included a source temperature of 150 °C, nitrogen desolvation, and cone gas flow of 800 and 50 L/h, respectively. A capillary and cone voltage of 1 kV and 16 V, respectively.

##### Standard solutions and calibration curves

The stock solution of ivacaftor was prepared in methanol while that of the internal standard lumacaftor was prepared in acetonitrile, both at a concentration of 1 mg/mL. ivacaftor stock solution was diluted into the working solutions range of (0.01 – 25 μg/mL) using methanol and lumacaftor was diluted to a working concentration of 2.5 μg/mL. Stock solutions of ivacaftor and lumacaftor were stored at – 80 °C and working solutions of both ivacaftor and lumacaftor were prepared freshly on the day of analysis. To construct the calibration curve, 100 μL of blank mouse plasma was spiked with 10 μL of each ivacaftor working solution (range: 0.01 – 25 μg/mL for a final concentration range of 0.001 – 2.5 μg/mL). ivacaftor was extracted from blank plasma that is spiked with the ivacaftor calibration standards to construct the calibration curve of ivacaftor in mice plasma. This ivacaftor plasma calibration curve was used to quantify ivacaftor concentrations in the plasma samples collected from mice in the in vivo pharmacokinetics study.

##### Calculation of limit of detection (LOD) and limit of quantification (LOQ)

The following equations were used to calculate the LOD and LOQ of the method according to the ICH guidelines (ICH harmonised tripartite guidelines).


Equation 1: $$LOD=\frac{3.3\times \text{Standard deviation of response}}{\text{Slope of the calibration curve}}$$

Equation 2: $$LOQ=\frac{10\times \text{Standard deviation of response}}{\text{Slope of the calibration curve}}$$

#### 2.2.6 Statistical analysis

All experiments were repeated 3 times. Microsoft Excel^®^ (2010) was used to analyze the in vitro drug release data, calculate the cumulative percentage of drug release, D10, D50, and D90, and perform span analysis for microparticle sizes. GraphPad Prism software (GraphPad, La Jolla, CA) was used for data visualization and statistical analysis. Multiple un-paired t-test with a two-stage step-up (Benjamin, Krieger, and Yekutieli) method was used to compare the in vitro release profile of formulation 1 vs every other formulation. Comparisons between each pair of formulations were performed separately. A p-value <0.05 was considered a statistically significant difference. Non-compartmental pharmacokinetics (NCA) analysis on in the vivo pharmacokinetic data was performed using PkSolver^®^ (an Excel Add-in)(Zhang, Huo, Zhou, and Xie 2010).

## 3. Results and Discussion

### 3.1 Development and characterization of ivacaftor-loaded PLGA microparticles

Ivacaftor-loaded microparticles were prepared using an oil-in-water single-emulsion solvent evaporation method. To obtain an ivacaftor-loaded microparticle formulation with the most optimum formulation characteristics such as an acceptable particle size for SC administration, highest DL and EE, and sustained drug release kinetics, we studied the effect of varying four formulation parameters on the produced microparticle formulation characteristics.

#### Microparticles size, shape, and surface morphology

**Figure 1:**
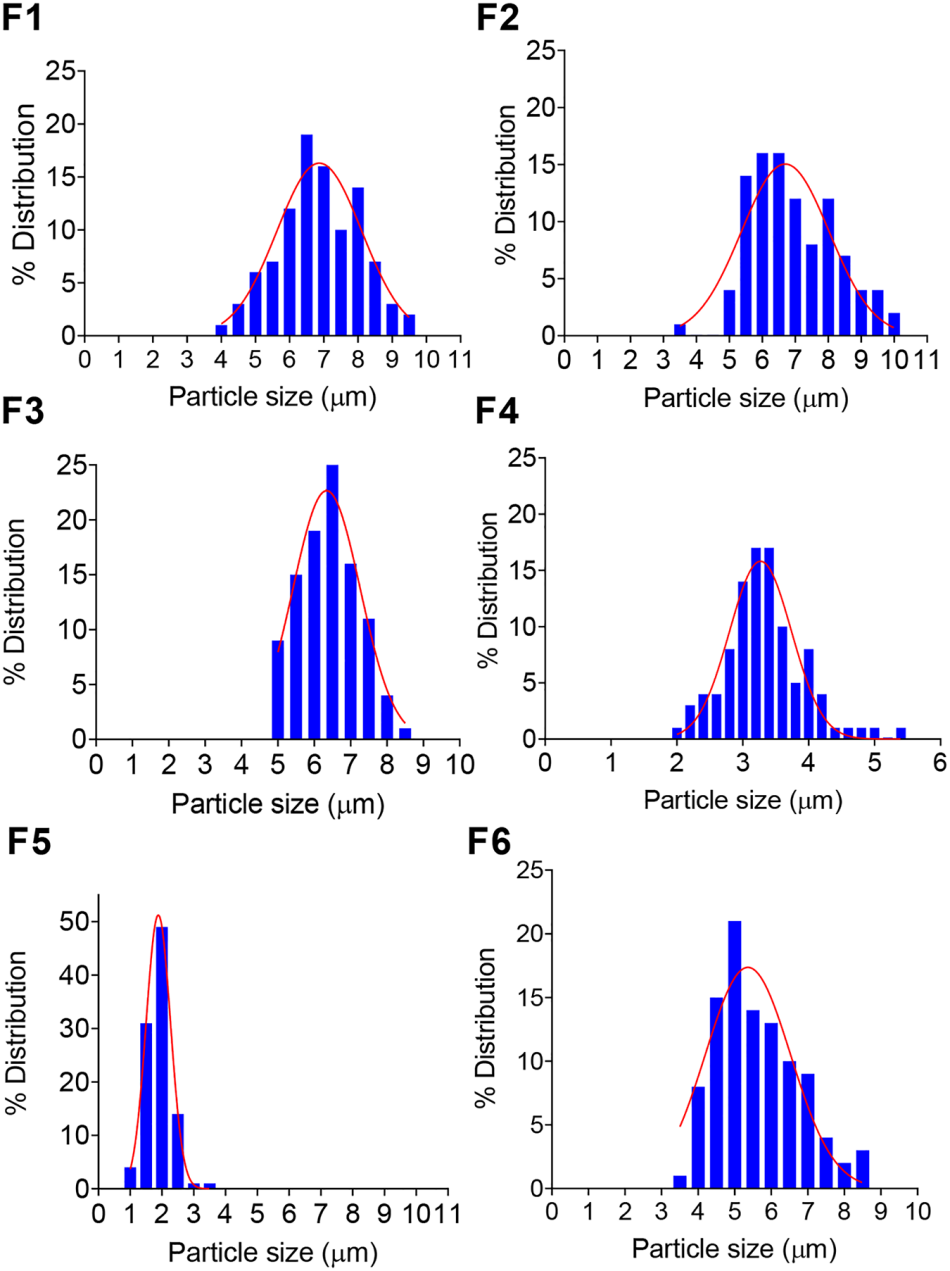
Particle size distribution histograms of the prepared microparticles (F1-F6) expressing a unimodal gaussian distribution (red solid line) which indicates a homogenous particle size distribution. Particle size was measured from scanning electron microscopy images using ImageJ^®^ software.

**Figure 2:**
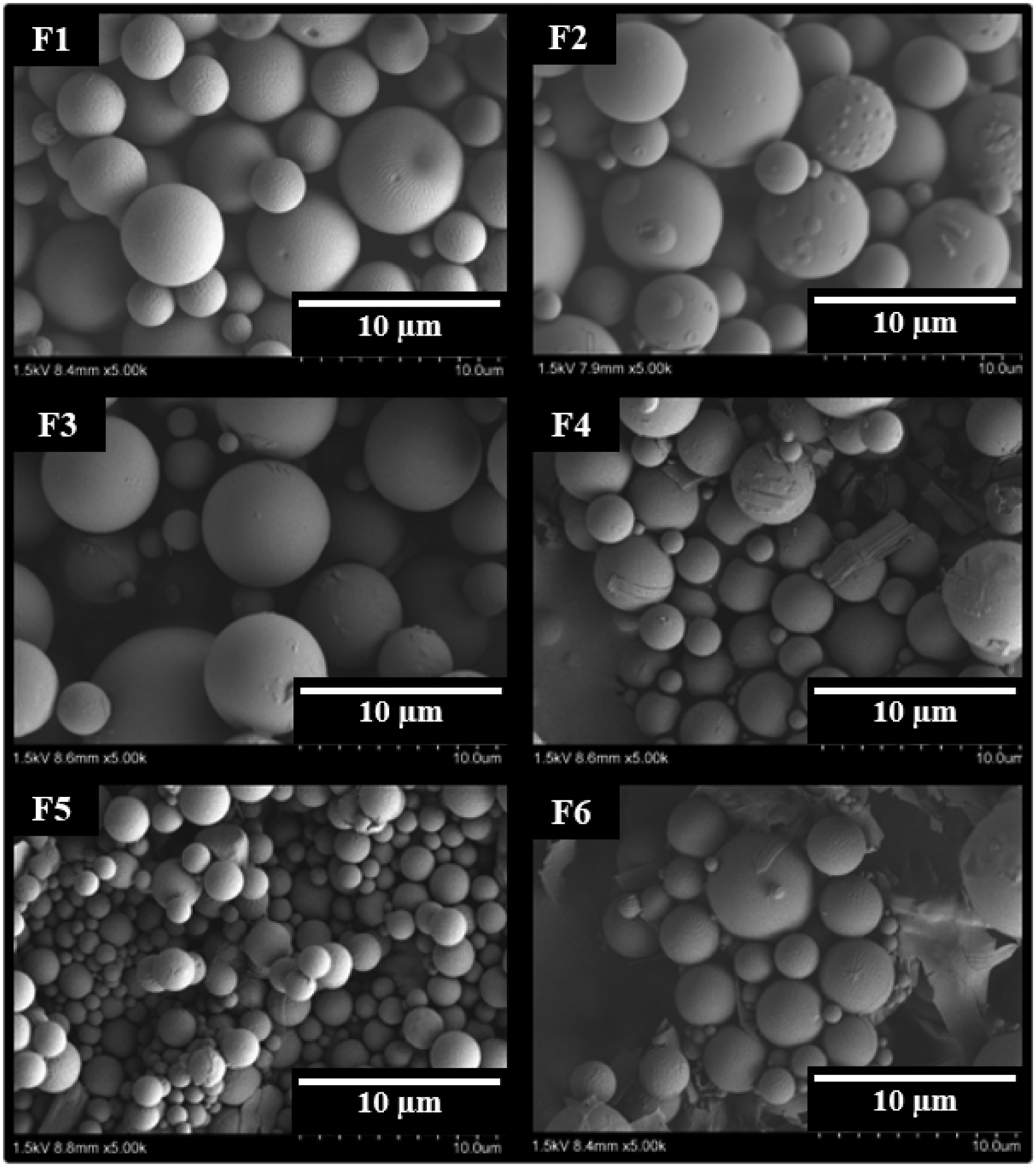
Representative scanning electron microscope (SEM) photomicrographs of ivacaftor-loaded microparticles (F1-F6) showing non-porous, spherical, and smooth surfaces with no unencapsulated drug crystals

The average diameter of the prepared ivacaftor-loaded microparticles ranged between 1.91 and 6.93 μm with narrow size distributions as demonstrated by span values ranging between 0.19 and 0.39. An acceptable microparticle size is reported to be that which can be injected through a 21 – 28-gauge needle or thinner (Park et al. 2019). For instance, Risperidal Consta^®^, a marketed microparticles formulation containing risperidone, which range from 25–180 μm is administered through a 21-gauge needle. Our formulation size range (1.91–6.93 μm) is well below this range, which makes the microparticles more convenient for patient administration. Figure 1 shows histograms of the particle sizes of each formulation as determined by ImageJ software utilizing 100 particles as described previously (Isely et al. 2019). The histograms showed unimodal homogenous particle size distribution indicating low variability in the particle size of the formulations. The numerical values of the average particle sizes are presented in Table 2. As shown in the SEM photomicrographs in Figure 2, the majority of the microparticles of F#1, 3, and 5 possessed smooth, non-porous surfaces with spherical shapes and no free drug crystals. In contrast, microparticles of formulations 2 and 4 show rectangular drug crystals deposited onto the surfaces of the microparticles. The effect of increasing the drug/polymer ratio from 10% (F1) to 20% (F2) was reflected in the presence of a few drug crystals on the surface, in addition to a bumpy surface, exhibited by F2 as compared to F1 which had a smooth surface. This could be attributed to the coexistence of ivacaftor in crystalline form along with its molecular form in F2 due to the marked hydrophobicity of ivacaftor (Mao et al. 2008; Naguib et al. 2009). Also, increasing the surfactant (PVA) concentration from 1% (F1) to 1.5% (F3) produced microparticles with a smaller particle size which could be related to the role of surfactant in reducing the interfacial tension between the aqueous and organic phases (Park et al. 2020b). A 1% w/v PVA concentration in F1 was sufficient to maintain the stability of the emulsion and produce particles with a desirable size distribution. In contrast, the average size of the microparticles was reduced from 3.32 μm to 1.91 μm by increasing the homogenization speed from 9500 rpm (F4) to 13500 rpm (F5). High homogenization speed results in the breakdown of the oil phase into smaller microdroplets due to the higher shearing forces provided by the higher stirring speeds (Joshi et al. 2013; Yang and Owusu-Ababio 2000a). The PLGA molecular weight also affected the size of the microparticles as a decrease of the PLGA molecular weight (in F6) resulted in a smaller particle size (5.64 μm) compared to 6.83 μm in F1 where a higher molecular weight PLGA was used. This can be explained by the higher viscosity of the oil phase when a higher molecular weight polymer is used which resists the shearing force during the emulsification process resulting in a larger average particle size (Xue 2004).

#### Differential scanning calorimetry and powder X-ray diffraction (PXRD)

**Figure 3:**
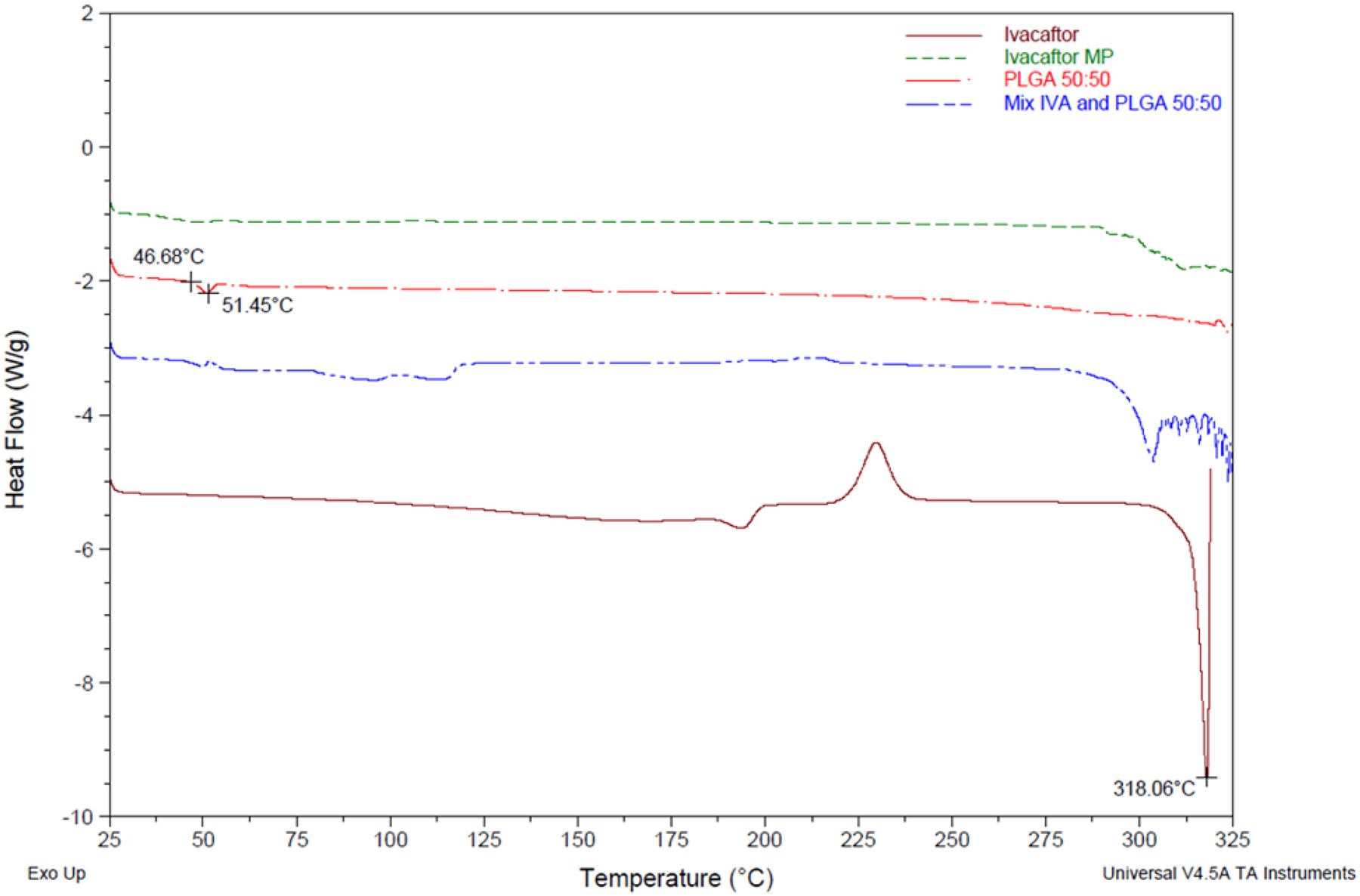
Differential scanning calorimetry (DSC) thermograms of ivacaftor, PLGA Resomer^®^ RG503H, ivacaftor PLGA microparticles, physical mixture of ivacaftor and PLGA. Ivacaftor shows a melting point at 318.06 °C which completely disappears in the microparticle formulation indicating that ivacaftor has lost its crystalline properties and is converted into an amorphous form which indicates that ivacaftor has been encapsulated into the microparticle formulation.

Solid-state characterization of pure ivacaftor, PLGA (Resomer^®^ RG503H), and their physical mixture and ivacaftor microparticles (F1) was performed using DSC and PXRD. Typically, when a crystalline drug is loaded into a microparticle formulation it loses its crystalline state and exists in a molecularly dispersed form due to the interactions with the polymer used (Naguib et al. 2009). Ivacaftor shows a melting point at 318.06 °C which completely disappeared in the microparticle formulation indicating that ivacaftor has lost its crystalline properties and is converted into an amorphous form which indicates that ivacaftor has been encapsulated into the microparticle formulation, as shown in Figure 3 (Park et al. 2020a). It is worth mentioning that various ivacaftor polymorphs have been reported in the literature, with different values for the melting point, ranging between 230 and 318 °C (Padiyath Mohammed Akbarali et al. 2017; Ram Thaimattam et al. 2017). The appearance of another endothermic peak at about 190 °C, in addition to an exothermic peak of crystallization at about 230 °C, may be explained on the basis of coexistence of more than one polymorph of ivacaftor in the batch that we used. The physical mixture shows signs of minimum but noticeable interaction between the drug and the polymer, as both the small endothermal peak at 190 °C, in addition to the exothermic peak at 230 °C, disappeared, while the major endothermic peak (melting) at 318 °C became smaller. These signs of partial interaction even in the physical mixture may be the result of the partial dissolution of the drug in PLGA when it is heated above its glass transition temperature (Tg, 51.5 °C), as the latter becomes softer above its Tg which allows ivacaftor molecules to diffuse within its matrix (Barghi, Vekalati, and Jahangiri 2023).

**Figure 4:**
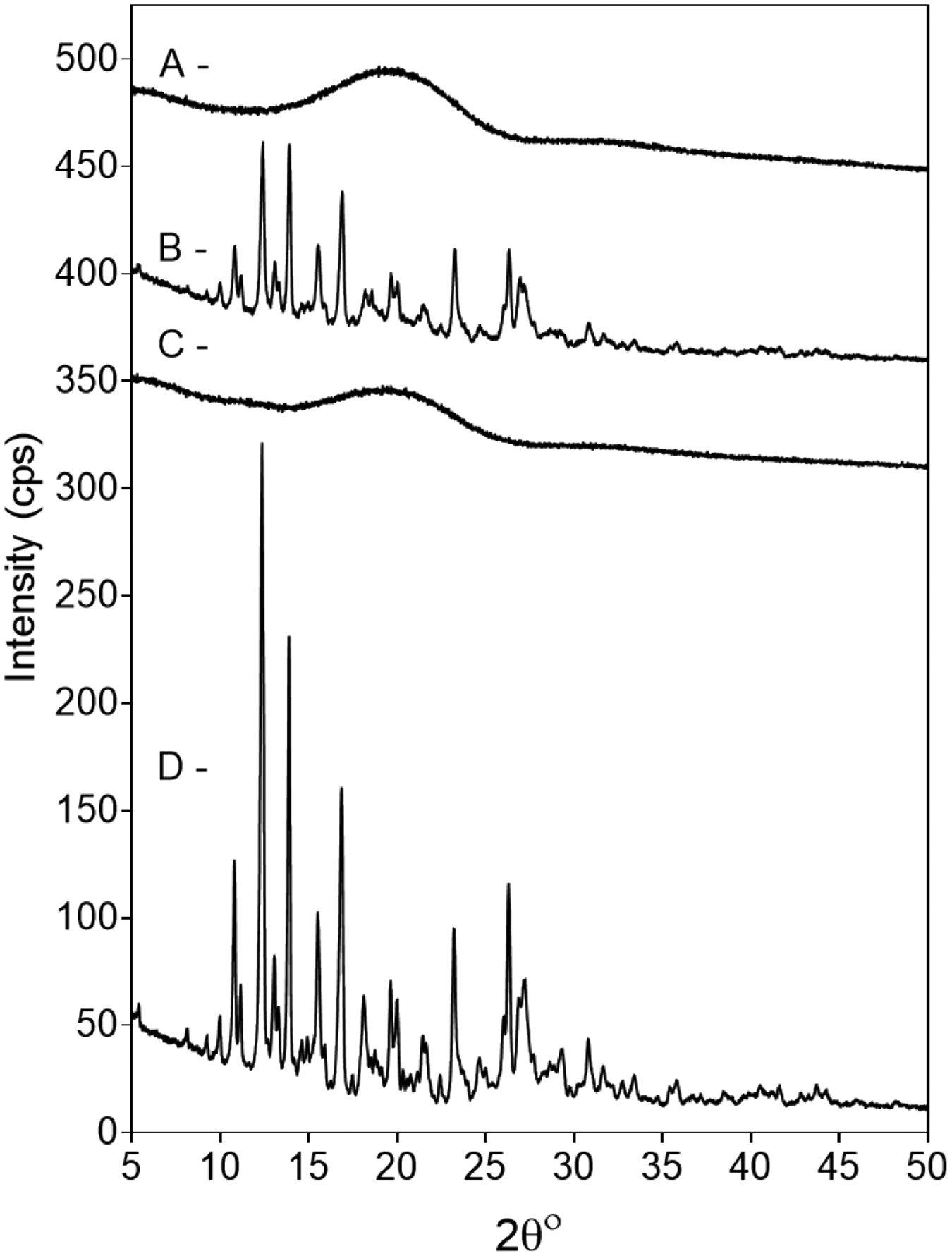
Diffractograms of A- ivacaftor microparticles, B- physical mixture of ivacaftor and PLGA RG503H, C- PLGA RG503H polymer, D- pure ivacaftor. The ivacaftor diffractogram shows clear and sharp crystalline peaks corresponding to its crystalline form which disappeared in the microparticle formulation indicating the conversion of ivacaftor into an amorphous state due to drug-polymer interaction because of its encapsulation inside the microspheres.

The loss of crystallinity was confirmed by the X-ray diffractogram in Figure 4 as diffractograms of pure ivacaftor and its physical mixture with PLGA show clear and sharp crystalline peaks corresponding to its crystalline form indicating that there was no interaction between the drug and the polymer upon simple mixing. However, the diffractogram of ivacaftor microparticles shows the disappearance of the crystalline peaks that correspond to the crystal structure of ivacaftor indicating that ivacaftor has been converted into an amorphous state due to possible interaction with the polymer. In contrat, as described above, this loss of crystallinity of ivacaftor in F1 due to encapsulation was incomplete in other formulations like F2 and F4. F2 had higher DL compared to F1, which may explain the coexistence of the drug in crystalline, along with amorphous forms in F2. This was evidenced by the SEM images which show drug rectangular crystals. Microparticles from F4 exhibit the same observation, despite the lower DL % in F4, compared to F1. The smaller particle size of F4 due to the faster homogenization speed may have changed the emulsion stability conditions of F4, and rendered the oil phase droplets less capable of accommodating drug molecules due to decreasing the path length drug molecules have to travel to diffuse out into the aqueous phase, resulting in deposition of drug crystals, which fail to dissolve in the external phase, on the surface of the microparticles.

### 3.2 Effect of formulation parameters on the drug loading, encapsulation efficiency, and the in vitro release profile.

The effect of varying different formulation parameters (drug/polymer ratio, surfactant concentration, emulsification speed, and PLGA molecular weight) on the DL, EE, and drug release profile of ivacaftor from the PLGA microparticles is shown in Table 2.

#### Effect of the drug/polymer ratio

**Figure 5:**
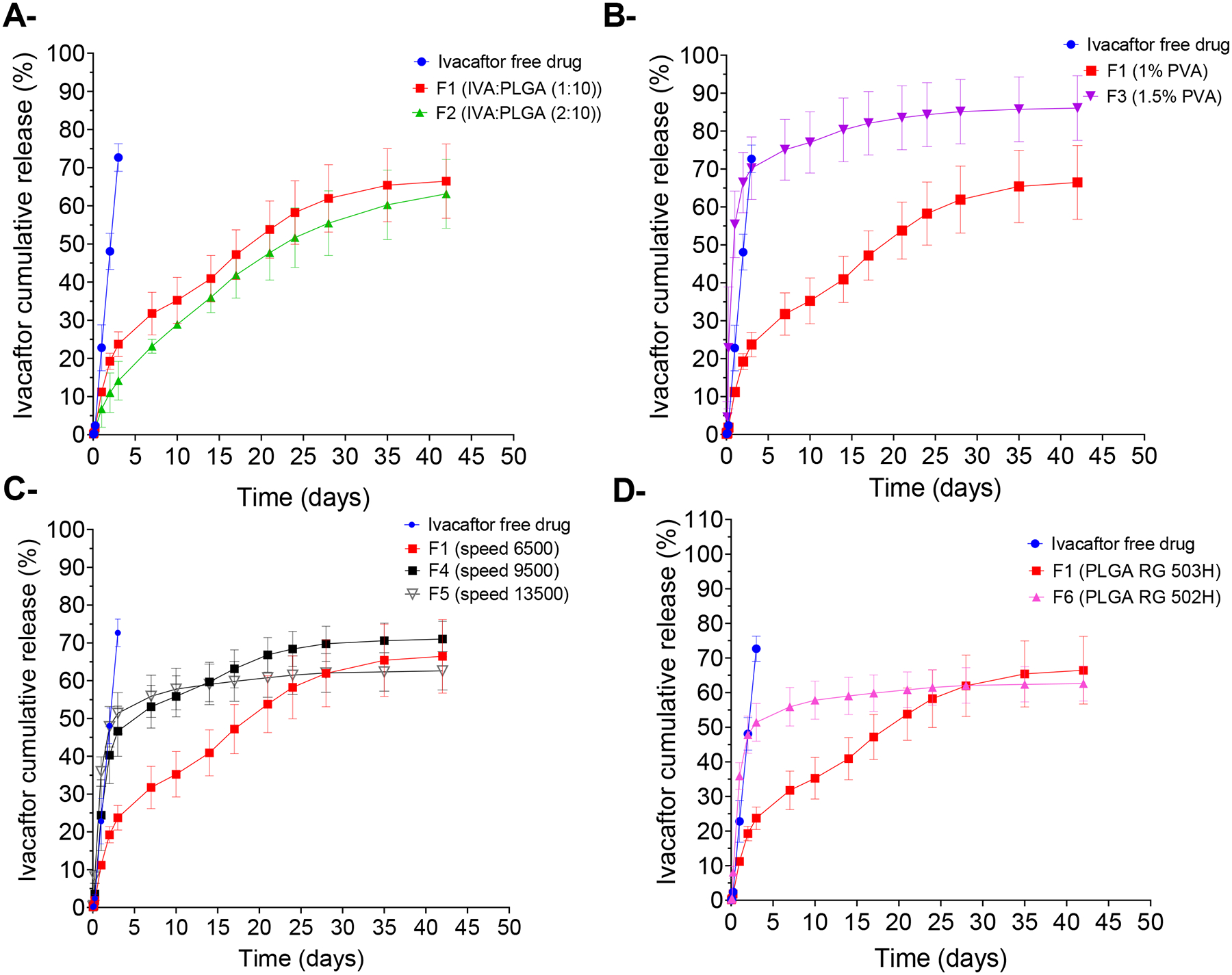
In vitro cumulative release profiles of the ivacaftor-loaded PLGA microparticles (F1- F6), showing the effect of (A) drug loading, (B) PVA surfactant concentration, (C) Homogenization speed, (D) PLGA molecular weight. Data is presented as mean ± SEM (n = 3).

To study the effect of the added amount of ivacaftor to the microparticles on DL, EE, and release profile of formulations, we increased the ivacaftor amount from 10% (F1) to 20% (F2) (1:10 to 2:10 Drug/polymer ratio, respectively) while keeping other formulation parameters constant. The average DL decreased from 12.13% ± 0.75 (F2) to 9.53% ± 1.91 (F1). This could be due to the lipophilic nature of the drug, as when more drug is initially added to the microparticle formulation, more drug stays in the inner oil phase rather than escaping to the external aqueous phase which results in higher D (Singh and Udupa 1997). The relatively higher DL F2 resulted in a lower average initial burst release of 14.14% ± 8.77 vs 23.73% ±5.6 in F1 after 3 days, and an overall relatively slower % cumulative drug release as shown in Figure 5A, although this difference was not statistically significant. Finally, in contrast to DL, F2 had a slightly lower average EE of 45.01% ± 5.96 in comparison to F1 with 76.25% ± 15.4. This can happen when the oil phase has reached its maximum saturation with the drug, so adding more of the drug would result in a lower EE (Cheng, Illum, and Davis 1998).

#### Effect of surfactant concentration

To investigate the effect of surfactant concentration on the microparticle formulation characteristics, F1 and F3 were prepared using 1% and 1.5% PVA, respectively. As described earlier, increasing the surfactant concentration has led to a decrease in particle size, as it improves the emulsion stability and reduces the interfacial tension between the aqueous and oily phases resulting in a smaller particle size which would affect their release profile. Since F3 (with a smaller particle size) has a larger specific surface area per unit volume, it had a stronger initial burst release and an overall faster cumulative release rate than F1 as shown in Figure 5B. After 3 days, 70% ±14.3 of ivacaftor was released from F3 where only 23.7 % ±5.6 were released from F1, and by 6 weeks, F1 showed 66.4% ±16.89 drug release compared to the 86.1% ±14.7 drug released from F3. The difference between the in vitro release profiles of both formulations was statistically significant (p <0.05) between day 1 and day10. Increasing the surfactant concentration also had an impact on lowering both %DL and %EE. F3 had an average %DL and %EE of 4.48% ±0.7 and 49.3% ±7.71 vs 9.53% ± 1.91 and 76.25% ± 15.4 for F1, respectively. The decrease in % DL and %EE upon increasing the surfactant concentration could be due to the smaller size of the generated microparticles (F3), which shortens the path of encapsulated drug to diffuse out before solidification of the microparticles takes place, besides the low tendency of ivacaftor to stay in the formulation due to the high surfactant concentration in the surrounding aqueous medium during the solvent evaporation step.

#### Effect of emulsification speed

We further studied the effect of the emulsification speed on the formulation characteristics by using 3 different speeds during microparticle preparation, namely 6500, 9500, and 13500 rpm for F1, F4, and F5, respectively. Higher emulsification speed generates a higher shear force which breaks down the oil phase into smaller droplets. When the small oil droplets evaporate, they end up forming microparticles with small particle sizes. Our data has shown that trend, where, F1 (with the lowest emulsification speed) had an average particle size diameter of 6.83 μm ±1.18 compared to 3.32 μm ±0.58 and 1.91 μm ±0.41 for F4 and F5, respectively. Smaller particle sizes are expected to have lower D and EE due to the smaller inside diameter of the particles where the drug can be loaded (Yang and Owusu-Ababio 2000b). F1 had average DL% and EE% of 9.53% ± 1.91 and 76.25% ± 15.4 compared to 3.91% ±0.37 and 43% ± 4.09 for F4, 2.59% ± 0.88 and 26.6% ± 9.71 for F5. Regarding the in vitro release profile, in smaller particles, the encapsulated drug needs to travel a shorter distance to the surface of the particles. In addition, the total surface area of particles exposed to the release medium is large. Both factors result in an overall fast cumulative release rate and a strong initial burst release with particles of a small size. Figure 5C shows the in vitro cumulative release profiles of F1, F4, and F5. After the first 3 days, 23.7% ±0.44 of ivacaftor were released from F1 while 29.13% ±22.41 and 46.63% ±11.53 were released from F4 and F5, respectively. By day 10, only 35.24% ±10.4 of ivacaftor were released from F1 while 54.1% ±15.3 and 55.8% ±9.4 were released from F4 and F5, respectively. Finally, at the end of the release study (6 weeks), 66.46% ±16.8 of ivacaftor have been released from formulation 1 vs 66.9% ±16.5 and 71.1% ±8.1 released from F4 and F5, respectively. However, these differences in the in vitro release profiles of F1 vs F4 and F5 were not statistically significant.

#### Effect of polymer molecular weight

Different molecular weights of PLGA polymer could be used in microparticle preparation. Here we used Resomer^®^ RG503H (F1) and RG502H (F6) with molecular weight ranges of 24,000 – 38,000 and 7,000 – 17,000 and inherent viscosities of 0.32 – 0.44 and 0.16 – 0.24 dL/g in chloroform, respectively. Decreasing the polymer molecular weight has resulted in microparticles with a smaller average diameter of 5.64 μm ± 1.12 in F6 compared to F1 (6.83 μm ± 1.18), as described above. As a result, F6 had a lower average %DL of 5.84 % ± 3.55 vs 9.53 % ± 1.91 for F1. This is because when a polymer of lower molecular weight is used, the inner oily phase has lower viscosity which offers less resistance to the shearing force during the emulsification process resulting in a smaller particle size. In addition, due to the lower viscosity of the oily phase, it becomes easier for the drug to escape to the external aqueous phase resulting in lower DL as seen in F6 (Gabor et al. 1999; Jaraswekin, Prakongpan, and Bodmeier 2007; Xue 2004; Yang et al. 2001). Changing the polymer molecular weight also had a notable effect on the drug release profiles as demonstrated in Figure 5D. The difference between the in vitro release profiles between both formulations was statistically significant between 6 hrs and 7 days after starting the experiment. F6 with lower molecular weight PLGA exhibited a stronger initial burst release and an overall faster release rate, especially during the first 4 weeks. After 3 days, 23.7% ± 5.6 and 51.4% ± 9.46 of the encapsulated drug was released from F1 and F6, respectively. This is due to the higher hydrophilicity of polymers with lower molecular weight resulting in faster degradation of the polymer and faster release (Han et al. 2016; Jaraswekin et al. 2007). This trend was reversed after the 30-day mark, where F1 had a slightly faster release than F6, releasing 66.4% ±16.9 and 62.6% ±8.7 by day 42, respectively. The reason for this could be due to the faster precipitation of the high molecular weight polymer used in F1 which results in the formation of pores in the matrix which could be responsible for the faster release in the last two weeks compared to F6 (Naguib et al. 2009).

### 3.3 In vitro release modeling

**Figure 6:**
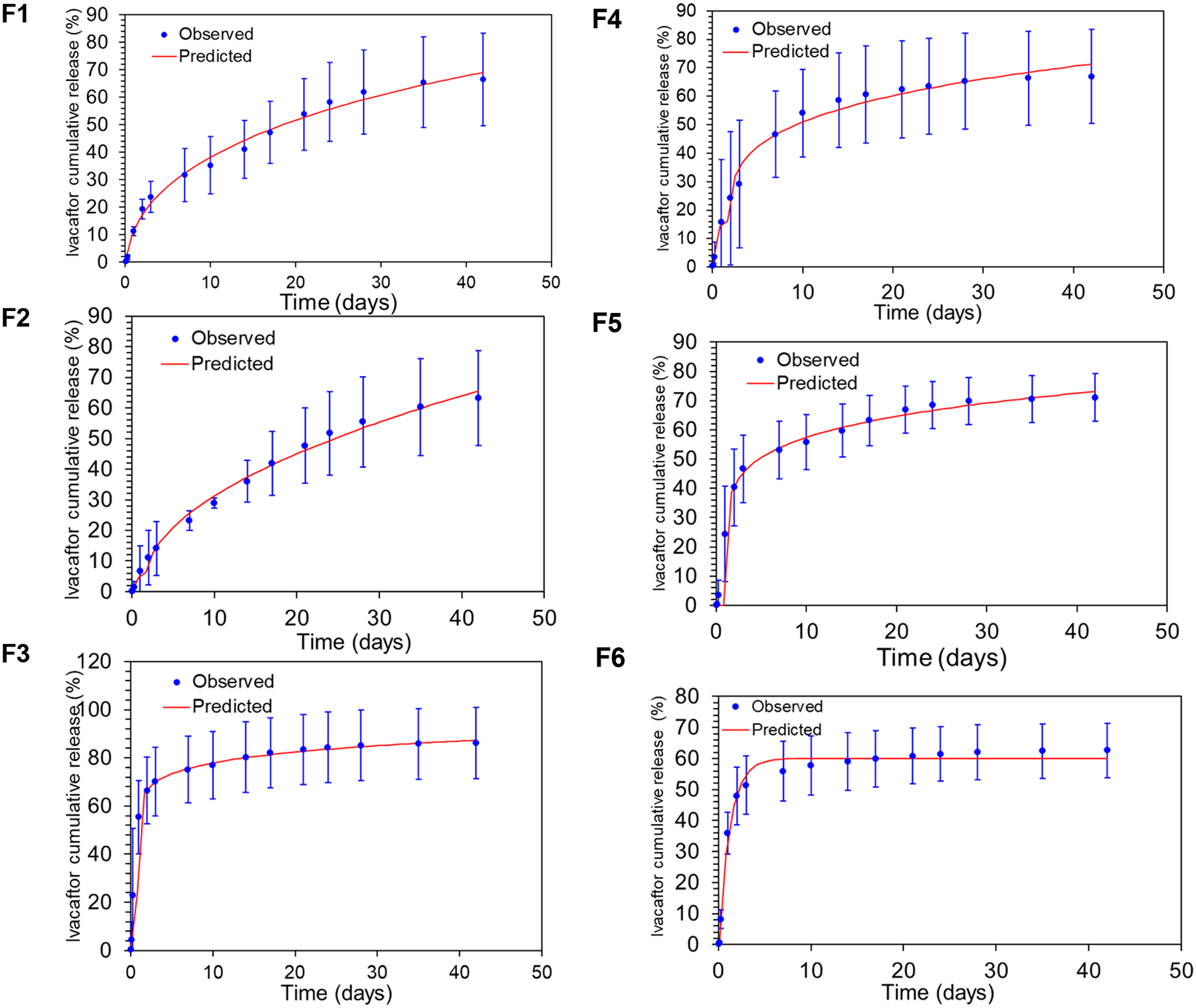
Best-fit release kinetic models to the in vitro release data of the ivacaftor-loaded PLGA microparticles. Observed data is represented as mean ±SD (n=3).

Modeling the in vitro release kinetics data of the ivacaftor-loaded PLGA microparticles using the DDsolver software showed that the 6 formulations followed either a “Baker-Lonsdale model, or a “Korsmeyer-Peppas model” or a “first-order” model. Where, F1 and F4 followed a Baker-Lonsdale model, F2, F5 and, F6 followed a Korsmeyer-Peppas and F3 followed a first-order model based on the model selection criteria (highest R-squared and lowest AIC). Figure 6 shows plots of the mean observed in vitro release data of the ivacaftor-loaded PLGA microparticles along with their best-fit models. Table 3 shows the results of fitting the in vitro release data to 5 release kinetics models (Zero order, first order, Korsmeyer-Peppas, Higuchi and Baker londsale) along with their goodness of fit parameters (AIC and R^2^ ). -

Baker and Londsale model is represented with the following equation:

$$3/2*\left[1-{\left(1-\text{F}/100\right)}^{\wedge }(2/3)\right]-\text{F}/100={\text{k}}_{\text{BL}}*\text{t}$$

Where F represents the fraction of the drug released over time (t), k_BL_ is the specific release rate constant.

Korsmeyer-Peppas is represented with the following equation:

$$\text{F}={\text{k}}_{\text{KP}}*{\text{t}}^{\wedge }\text{n}$$

Where F represents the fraction of the drug released over time (t), k_KP_ is the release rate constant, n represents the diffusional coefficient correlated to the release mechanism.

Finally, the first-order model is represented with the following equation:

$$\text{F}=100*\left[1-\mathrm{Exp}\left(-{\text{k}}_{1}*\text{t}\right)\right]$$

Where, F represents the fraction of the drug released over time (t), and K_1_ represents the first-order release rate constant.

The use of mathematical models to fit data obtained from the in vitro drug release experiments of a controlled-release drug delivery system helps determine whether the release is driven primarily by diffusion, matrix erosion, or a combination of the two. This helps optimize the design of the drug delivery system by modulation and careful selection of type and amount of the API, and the shape, size, and matrix composition of the carrier system, to achieve a specific drug release profile in order to ultimately meet the therapeutic goals of the treatment regimen (Peppas and Narasimhan 2014; Siepmann and Peppas 2001).

Thes best fit model to the experimental in vitro release data was selected based on the highest and the lowest, R-squared and AIC values, respectively, as presented in Table 3.

The in vitro release data of formulations 1 and 4 followed a typical bi-phasic diffusion-erosion mechanism of PLGA polymer which was best described by Baker-Londsale model equation. This model is also known as a spherical matrix model in which the drug release is driven by combined diffusion-erosion mechanism, where, first, the drug near the surface of the microsphere diffuses to the outer matrix. This is followed by the release of the drug near the core of the microsphere through pores and/or capillaries that are created in the polymer matrix as a result of its hydrolytic erosion (Naguib et al. 2009).

In contrast, formulations 2, 5 and 6 showed a rapid initial burst release followed by a slower release phase. The in vitro release data from these formulations were best fitted by the model equation developed by Korsmeyer-Peppas. This is a semi-empirical model, that provides an exponential relationship involving the drug release as a function of time.

The exponential diffusional coefficient (n) in formulations (F2 – F5) was less than 1 and ranged from 0.22 – 0.47. This is because, following the initial rapid burst release of the drug, the curve slowly plateaus but doesn’t reach a constant plateau phase (Costa, Manuel, and Lobô´´ 2001) . Formulations 4 and 5 in particular, showed release kinetics more consistent with simple Fickian diffusion, especially in the first 48 hours of the release experiment where about 45–50% of the drug cargo was released. This is due to the relatively large surface area of the microparticles during the release experiment as the particle sizes of these two formulations are significantly smaller than those of Formulations 1–3 (Table 2).

Formulations 1 and 2 show release kinetics more consistent with combined diffusion/erosion, especially Formulation 1, as the release slowed between 7–14 days, which is consistent with the end of diffusion phase, then the release rate started to increase again after the 2nd week, which may mark the beginning of matrix erosion. Formulation 2 release profile does not fit Fickian diffusion, as reflected in the n value (>0.45). In fact, the release profile of Formulation 2 leans more towards a zero order model, especially during the first three weeks, however, due to the relatively lower EE% and yield %, this formulation was not chosen for in vivo experiments.

Finally, formulation 3 showed a diffusion-driven first-order release behavior that was characterized by an initial burst release, due to the presence of a large amount of encapsulated drug towards the periphery. After the burst period, the release rate then slowed down, in a direct proportionality with the remaining amount to be diffused, which is a typical first order behavior. The existence of a large amount of the drug towards the periphery can be explained by the presence of high surfactant concentration (1.5% PVA) in the surrounding aqueous medium during the particles’ preparation process. Since ivacaftor is hydrophobic in nature, the higher surfactant concentration in the surrounding aqueous medium resulted in attracting the drug towards the surface of the particles rather than staying in the core during the solvent evaporation/solidification process.

As mentioned above, the exponential diffusional coefficient (n) of the 6 formulations varied according to whether their release primarily followed Fickian diffusion (n<0.45) or non-Fickian transport (n = 0.45 – 0.89) (Zhan et al. 2019; Zhu, Long, and Shi 2023). The in vitro release from all formulations primarily followed a Fickian diffusion mechanism (n < 0.45) except for formulations 1 and 2 where the in vitro release was controlled by a non-Fickian transport mechanism.

These mathematical models can be used to predict the drug release at different time points beyond the in vitro release experiments that were performed. This could be useful since it will save the time needed to screen other formulations and to run more in vitro release experiments which will expedite the formulation development process and help reach the desired formulation parameters that can achieve the targeted drug release profiles (Zhan et al. 2019; Zhu et al. 2023). Even though the models represented herein revealed some important information about the mechanism of drug release from the formulation, usefulness of their application in optimizing the preparation method, especially in large scale production, remains limited. More comprehensive evaluation of other formulation parameters like different surfactant concentrations, emulsification speeds, drug/polymer ratios, polymer molecular weights, volumes of the organic and aqueous phases, concentrations of the drug and the polymer in the organic phase, and the preparation method will undoubtedly improve the prediction ability of these models and maximize the benefit of their application.

### 3.4 In vivo pharmacokinetics

**Figure 7:**
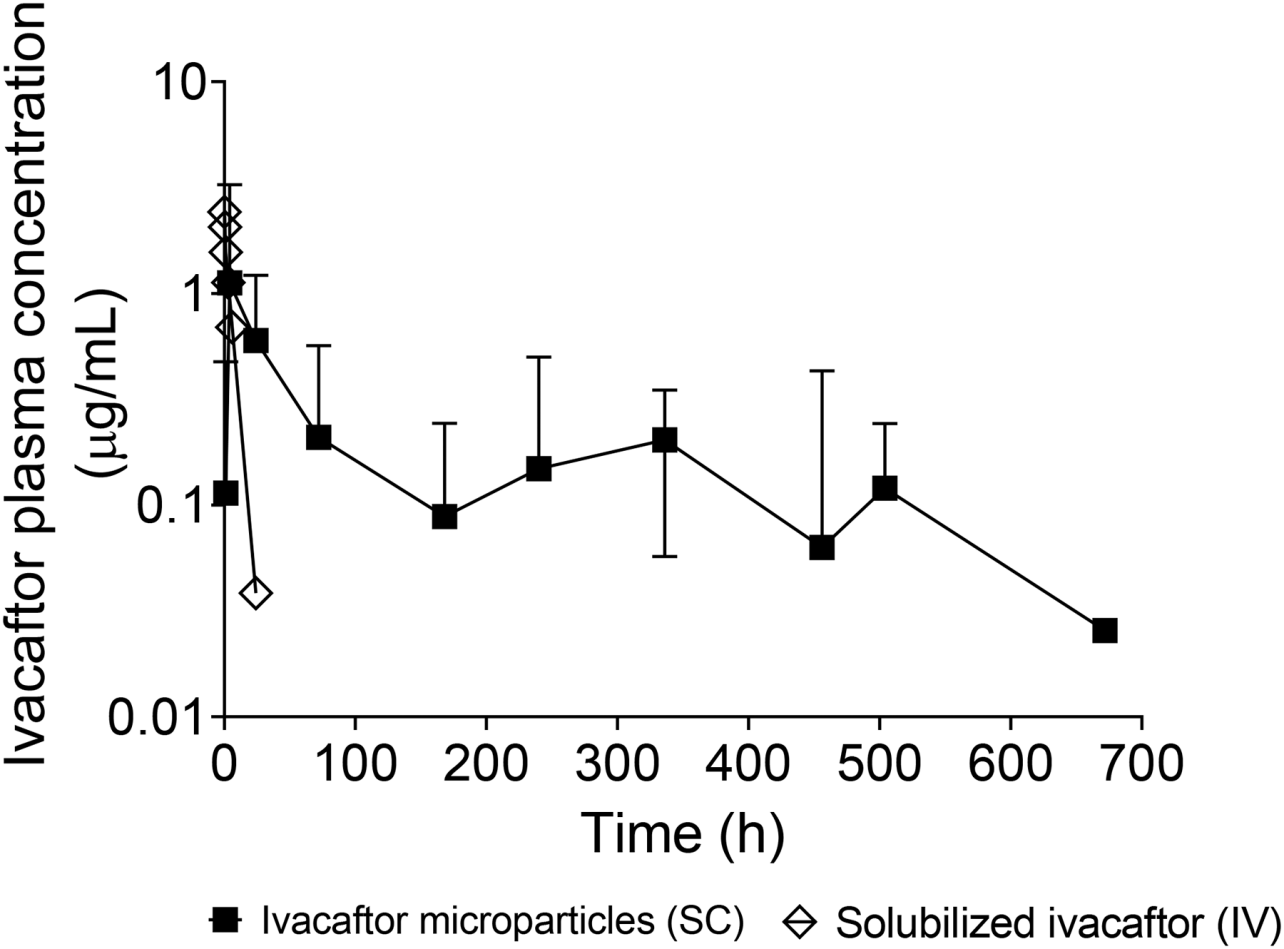
Mean plasma concentration-time profile of ivacaftor following the SC administration of ivacaftor-loaded PLGA microparticles (formulation 1) to mice (n=30) at a dose of 50 mg/kg vs IV administration of soluble ivacaftor (in a 10% Tween 80 in PBS, pH = 7.4 vehicle) to mice (n=21) at a dose of 5 mg/kg. Error bars represent the standard deviation (±SD, n =3).

Based on the characterizations of the prepared ivacaftor-loaded microparticles (particle size, %DL, %EE, and in vitro drug release profiles), F1, with %DL of (8.25 ± 3.73), %EE of ( 90.7 ± 41.1), a slow overall in vitro release profile and appropriate particle size (6.83 μm ± 1.18) that makes it suitable for SC injection (Park et al. 2019) was selected for further in vivo pharmacokinetics study in mice. The plasma concentration-time profile of ivacaftor following the IV and SC administration of 5 mg/kg solubilized ivacaftor and 50 mg/kg ivacaftor-loaded microparticles (F1), respectively, in mice are shown in Figure 7. The initial burst release of ivacaftor from the microparticles at 4 h (approx. 1 μg/mL) is approximately 10 times that of the average steady-state plasma concentration (approx. 0.1 μg/mL) later in time. This difference between initial burst-release plasma concentration and steady-state plasma concentration is common after the SC administration of microparticles and has been reported in the literature (Park et al. 2019). Sustained mice plasma levels of ivacaftor were observed for up to 28 days (4 weeks) following the SC administration of ivacaftor microparticle formulation (F1) with concentrations ranging from 0.02 – 1.12 μg/mL. This is in contrast to the rapid elimination of ivacaftor following its IV administration to mice where the last detectable plasma concentration was after only 1 day of administration. Sustained plasma levels of ivacaftor (up to 4 weeks) following the SC administration of the microparticles was achieved, in comparison to the IV administered solubilized ivacaftor (up to 24 h) (71.6 vs 12.3 μg/mL.h for ivacaftor microparticles and solubilized ivacaftor, respectively). It is worth noting that this data is not dose-normalized and the SC dose of ivacaftor microparticles was 10 times higher than that of the IV administered solubilized ivacaftor (1 vs 0.1 mg). This is because when the drug is encapsulated into polymeric microparticles it can be safely injected at higher doses via the SC route than it would be when administered directly into the blood stream via the IV route. As a result, the dose of the IV administered ivacaftor was lowered to 0.1 mg to avoid any toxic reactions.

Absolute bioavailability was calculated using the following equation:

$$\text{Absolute bioavailbility}=\frac{\left(\frac{\text{AUC microparticles}}{\text{Dose microparticles}}\right)}{\left(\text{AUC IV}\right)}$$

Absolute bioavailability of ivacaftor following the sub cutaneous administration of the microparticles (F1) to mice was 0.58.

In a clinical trial, the plasma levels of ivacaftor were between 0.5 – 1 ug/ml following the administration of 150 mg ivacaftor to healthy volunteers (Liddy et al. 2017). In our study, the mice’s plasma levels of ivacaftor were between 0.02 – 1.12 μg/ml following the administration of 1 mg ivacaftor microparticle formulation via the SC route. These results indicate that the SC administration of ivacaftor microparticles to mice provides plasma levels of ivacaftor that are within its therapeutic range and can potentially be scaled up for human administration. Single SC administration of ivacaftor microparticles (F1) can potentially eliminate the need for frequent daily administration of ivacaftor to treat CF by providing sustained plasma levels over an extended period of time (28 days).

### 3.5 In vitro in vivo correlation

**Figure 8:**
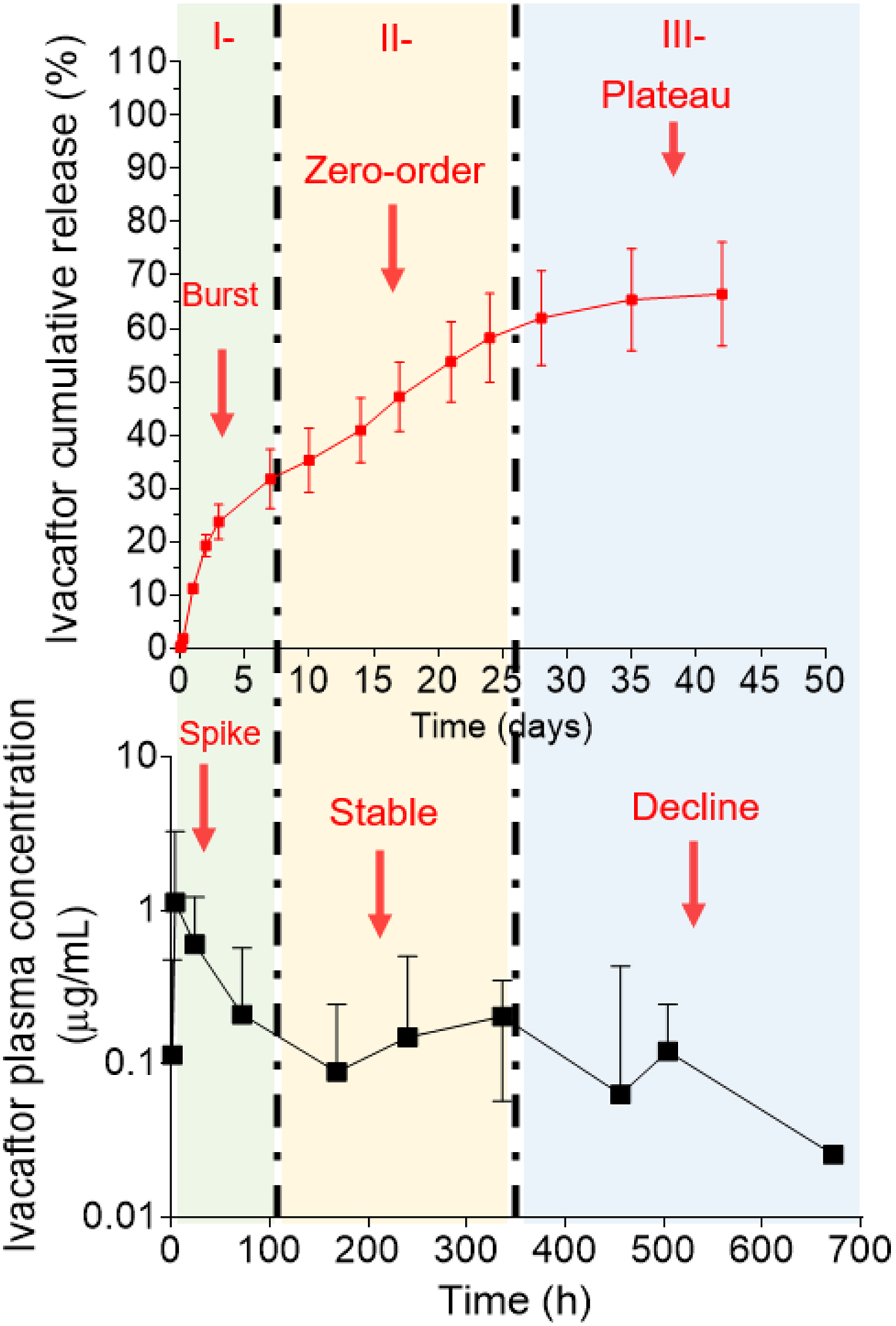
In vitro in vivo correlation of ivacaftor-loaded PLGA microparticles formulation (1)

Figure 8 depicts the relationship between the cumulative in vitro release of ivacaftor in aqueous media to its in vivo plasma concentrations following the administration of ivacaftor microparticles to mice via the SC route. Ivacaftor release from the microparticles both in vitro and in vivo is characterized by 3 phases due to the combined diffusion-erosion mechanism of the PLGA polymer. Phase 1 shows an initial burst release in vitro (5 – 7 days) primarily due to the diffusion of the drug that is near or at the surface of the particles. This burst release was correlated in vivo with a spike in ivacaftor’s plasma concentrations in mice (2 – 4 days). This phase was followed by a more constant (near zero-order) in vitro release (day 7 – day 25) due to the time it takes for the acidic build up that is resulting from the hydrolysis of the PLGA polymer. This was correlated in vivo to a more constant/stable ivacaftor plasma concentrations (10 days). Finally, the last phase (phase 3) is characterized by a modest increase in the in vitro release (day 25 – 35) due to the beginning of erosion of the PLGA polymer and then a plateau of the drug concentrations (last 5 days) due to the complete erosion of the PLGA polymer. This was correlated in vivo to an overall decline in the ivacaftor’s plasma concentrations in the last two weeks. This is likely because the amount released of ivacaftor during that time after the polymer erosion was only about 10% more which wasn’t enough to cause a significant increase in the drug’s plasma concentrations in vivo due to the elimination of the drug at the same time at a higher rate than that of its release from the microparticle at this phase. Finally, even though more accurate estimation of PK data relevant to free ivacaftor will require the use of more time points, we believe the reported PK data is sufficient to provide an estimate prediction of which model fits better with this data, and also sufficient to provide a legitimate control to which SC data can be compared.

## 4. Conclusion

Ivacaftor (VX-770) is a member of a novel class of drugs used to treat CF called CFTR modulators. It has been marketed by Vertex Pharmaceuticals since 2012 in an oral tablet form under the brand name Kalydeco^®^ and to achieve therapeutic plasma levels in people with CF, Kalydeco^®^ must be administered twice daily at a dose of 150 mg. Frequent daily administration is known to cause less compliance and adherence of patients to the treatment regimen. The finding of this study provided a potential solution for this problem by encapsulating ivacaftor into an injectable long-acting PLGA-based microparticle formulation using a single oil-in-water emulsion solvent evaporation method. By varying some of the formulation parameters (Drug: polymer ratio, surfactant concentration, emulsification speed, and polymer molecular weight), we developed ivacaftor-loaded microparticles (F1) with optimum characteristics. Results showed an optimum particle size of the prepared microparticles suitable for SC injection and a good DL that allows the injection of an appropriate amount of particles to achieve the target dose. In addition, the 90% EE may indicate that this formulation has the potential of cost-effective scaling up with minimum drug loss. The in vitro release profile of the formulation showed the typical biphasic release pattern observed with PLGA-based microparticles, with a small initial burst release and an overall slow cumulative release. When we injected the microparticle formulation SC into mice, ivacaftor had steady plasma levels that were detectable for up to 28 days. By comparing the SC-injected ivacaftor-loaded microparticle (F1, 1 mg ivacaftor) with an IV-injected soluble ivacaftor (0.1 mg ivacaftor), it showed longer/extended plasma exposure of the drug (up to 4 weeks). These microparticles provide, for the first time, a potential clinically translatable, sustained-release injectable formulation that can eliminate the need for the frequent daily administration of oral ivacaftor which will thus improve patient compliance and adherence with the treatment regimen and ensure positive CF treatment outcomes.

## Supplementary Material

1

2

## Figures and Tables

**Table 1:** Different formulation parameters were studied in the preparation of ivacaftor-loaded PLGA microparticles.

	Formulation Parameters			
Formulation	Ivacaftor amount (mg)	PLGA type	PVA concentration (%)	Homogenization speed (rpm)
**F1**	5	Resomer^®^RG 503 H	1	6500
**F2**	10	Resomer^®^RG 503 H	1	6500
**F3**	5	Resomer^®^RG 503 H	1.5	6500
**F4**	5	Resomer^®^RG 503 H	1	9500
**F5**	5	Resomer^®^RG 503 H	1	13500
**F6**	5	Resomer^®^RG 502 H	1	6500

**Note: Resomer**^®^ **RG 503 H:** PLGA (50:50) Mw (24,000–38,000) acid terminated,

**Resomer**^®^ **RG 502 H:** PLGA (50:50) Mw (7,000–17,000) acid terminated,

**PVA:** Polyvinyl alcohol

**Table 2:** Characteristics of the prepared ivacaftor-loaded PLGA microparticle formulations

	Drug Loading (DL) % (w/w ± SD)^1^	Encapsulation efficiency (EE) % (w/w ±SD)^1^	Yield% (w/w ±SD)^1^	D_10_ (μm)	D_50_ (μm)	D_90_ (μm)	Average diameter (μm±SD)^1^	Span value
F#								
**F1**	9.53 ± 1.91	76.25 ± 15.4	72.72 ± 2.91	5.26	6.83	8.3	6.83 ± 1.18	0.28
**F2**	12.13 ± 0.75	45.01 ± 5.96	61.67 ± 4.62	5.45	6.71	8.61	6.93 ± 1.31	0.35
**F3**	4.48 ± 0.7	49.3 ± 7.71	61.4 ± 10.5	5.38	6.36	7.38	6.38 ± 0.77	0.19
**F4**	3.91 ± 0.37	43 ± 4.09	70.3 ± 9.15	2.6	3.28	3.99	3.32 ± 0.58	0.26
**F5**	2.59 ± 0.88	26.6 ± 9.71	63.6 ± 15.3	1.47	1.9	2.43	1.91 ± 0.41	0.36
**F6**	6.61 ± 5.26	33.2 ± 7.27	45.3 ± 5.26	4.26	5.51	7.19	5.64 ± 1.12	0.39

1 Mean ± standard deviation (SD, n=3)

**Table 3:** Results of fitting the in vitro release data of ivacaftor-loaded PLGA microparticles to 5 in vitro release kinetics models (A) along with goodness of fit parameters (B).

A-											
	Zero-order		First-order		Higuchi		Korsemeyer-Peppas			Baker-Londsale	
F#	*K_o_*	*R* ^*2*^	*K_1_*	*R* ^*2*^	*K_H_*	*R* ^*2*^	*n*	*K_kp_*	*R* ^*2*^	*K_BL_*	*R* ^*2*^
1	2.1	0.74	0.038	0.91	11.2	0.98	0.47	12.5	0.98	0.003	0.98
2	1.9	0.77	0.03	0.87	9.97	0.94	0.56	8.57	0.97	0.002	0.93
3	3.12	0.01	0.694	0.67	17.9	0.31	0.22	43.4	0.81	0.01	0.57
4	2.33	0.332	0.06	0.72	12.9	0.8	0.38	20.2	0.9	0.005	0.87
5	2.5	0.16	0.07	0.66	13.9	0.77	0.31	25.8	0.9	0.006	0.87
6	2.28	0.01	0.06	0.2	13.1	0.5	0.24	29.7	0.82	0.005	0.65
B-											
	Zero-order		First order		Higuchi		Korsemeyer-Peppas			Baker-Londsale	
F#	*AIC*		*AIC*		*AIC*		*AIC*			*AIC*	
1	116		98.4		76.5		76			73.5	
2	106.3		83.2		90.9		78.4			93.6	
3	151		118.2		136.3		120.4			129.3	
4	130.6		113.9		109.9		105.5			104.9	
5	136.8		123.5		116.2		105.6			107.6	
6	140.7		132.4		125.5		111.4			120	

## Data Availability

Available upon request
