# Supplementary material for: INJECTABLE LONG-ACTING IVACAFTOR-LOADED POLY (LACTIDE-CO-GLYCOLIDE) MICROPARTICLE FORMULATIONS FOR THE TREATMENT OF CYSTIC FIBROSIS: IN VITRO CHARACTERIZATION AND IN VIVO PHARMACOKINETICS IN MICE
Source: Int J Pharm. Author manuscript; Available in PMC 2024 Feb 5. (PMC10843602; doi:10.1016/j.ijpharm.2023.123693)
Supplement: 2 [file NIHMS1954829-supplement-2.pdf]

Journal Pre-proofs

Supplementary section

Analytical methods

HPLC-UV

Supplemental Figure 3 shows calibration curve and representative HPLC-UV chromatograms of ivacaftor in aqueous samples. The calibration curves had an r-squared value of 0.999 and the retention time of ivacaftor was 6.9 min. **Supplemental Table 2** shows a summary of the HPLC-UV method parameters used for the detection of ivacaftor. Ivacaftor was successfully extracted and measured, and the sensitivity was quite high with detection in the microgram range (0.1 µg/mL). This demonstrates that the in vitro drug samples can be successfully analyzed using this analytical technique.

LC-MS/MS

LC-MS/MS was used to analyze the samples collected from the in vivo studies. **Supplemental Figure 4** shows representative LC-MS/MS chromatograms of ivacaftor and the internal standard lumacaftor after extraction from mice plasma in addition to blank plasma. The plasma standard curve of ivacaftor had good reproducibility in the range of 0.001 – 2.5 µg/mL with %RSD values ranging from 0.094 – 5.063 %. The standard curve was fitted using a liner regression equation (Y=4.819 x+0.3143, r-squared = 0.988), where Y is the peak area ratio of ivacaftor/lumacaftor (the internal standard) and x is ivacaftor's concentration. The retention times of ivacaftor and lumacaftor were 4.97, and 4.12 min, respectively. **Supplemental Tables 3 and 4** show a summary of the optimized liquid chromatography (LC-MS/MS) parameters for the detection of ivacaftor and the internal standard lumacaftor. **Supplemental Table 5** shows the calibration curve of ivacaftor along with the %RSD values and the LOD and LOQ values.

Supplemental Table 1: Equations of the in vitro release models used to fit the in vitro release kinetics data.

	**Equations**
**Zero-order**	$F=k_0*t$
**First order**	$F=100*[1-Exp(-k_1*t)]$
**Higuchi**	$F=k_H*t$^0.5
**Korsemeyer-Peppas**	$F=k_{KP}*t$^n
**Baker-Londsale**	3/2*[1-(1-F/100)^(2/3)]-F/100=$k_{BL}*t$

Journal Pre-proofs

Supplemental Table 2: HPLC-UV method parameters for the detection of ivacaftor in aqueous samples and following extraction from mice plasma.

**Column**	Reversed-phase Waters® Symmetry C_{18} column (5 µm pore size, 4.6 mm ID. × 150 mm)
**Mobile phase**	Acetonitrile: 0.1% TFA in water (60:40, v/v)
**Flow rate**	1 ml/min
**Injection volume**	50 uL
**Wavelength**	309 nm
**Range**	0.1 – 50 ug/mL

Journal Pre-proofs

Supplemental Table 3: Summary of the liquid chromatography (LC) conditions for the detection of ivacaftor and the internal standard lumacaftor using LC-MS/MS

**System**	Waters® Acquity H-class ultra-performance liquid chromatography (UPLC)
**Column**	Agilent® RRHD Eclipse Plus C8 column (2.1 ID x 100 mm, 1.8 um pore size)
**Mobile phase**	(A) water with 0.1 % trifluoroacetic acid v/v and<br>(B) acetonitrile
**Flow rate**	0.2 mL/min
**Injection volume**	20 uL
**Range**	0.001 – 2.5 ug/mL

Journal Pre-proofs

Supplemental Table 4: Summary of the LC/MS-MS optimized parameters for the detection of ivacaftor and the internal standard lumacaftor.

Analyte	Q1 (m/z)	Q3 (m/z)	CE (V)	Retention time (min)
**Ivacaftor**	393.18	172.07	28	5
**Lumacaftor (IS)**	453.02	131.04	40	4.2

Journal Pre-proofs

Supplemental Table 5: Ivacaftor LC/MS-MS calibration curve responses (peak area ratio) along with the calculated %RSD.

Ivacaftor concentration (µg/mL)	Peak area ratio			% RSD
0.001	0.069692257	0.066163532	0.073220981	5.063
0.005	0.194339359	0.185393091	0.203285626	4.603
0.010	0.111343186	0.106973809	0.115712563	3.924
0.025	0.215367508	0.205624997	0.225110018	4.524
0.050	0.413569416	0.40677853	0.420360301	1.642
0.100	0.699295667	0.673760978	0.724830356	3.651
0.25	1.708075679	1.658282733	1.757868624	2.915
0.5	3.231931246	3.163324792	3.300537699	2.123
1	5.992707119	5.910563937	6.074850302	1.371
2.5	11.90715499	11.89592449	11.91838548	0.094

LOD: 0.0027 µg/mL

LOQ: 0.0083 µg/mL

Journal Pre-proofs

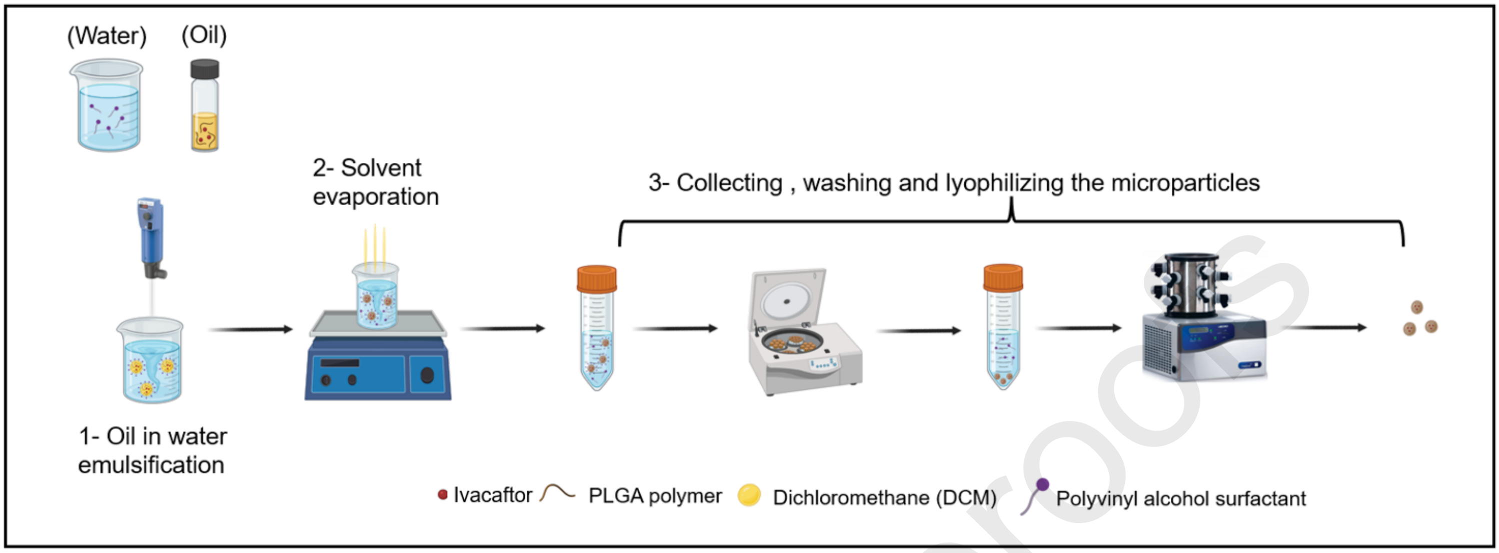

Supplemental Figure 1 Single emulsion (oil-in-water) solvent evaporation technique for the fabrication of Ivacaftor-loaded PLGA microparticles. Images created by Biorender.com.

Journal Pre-proofs

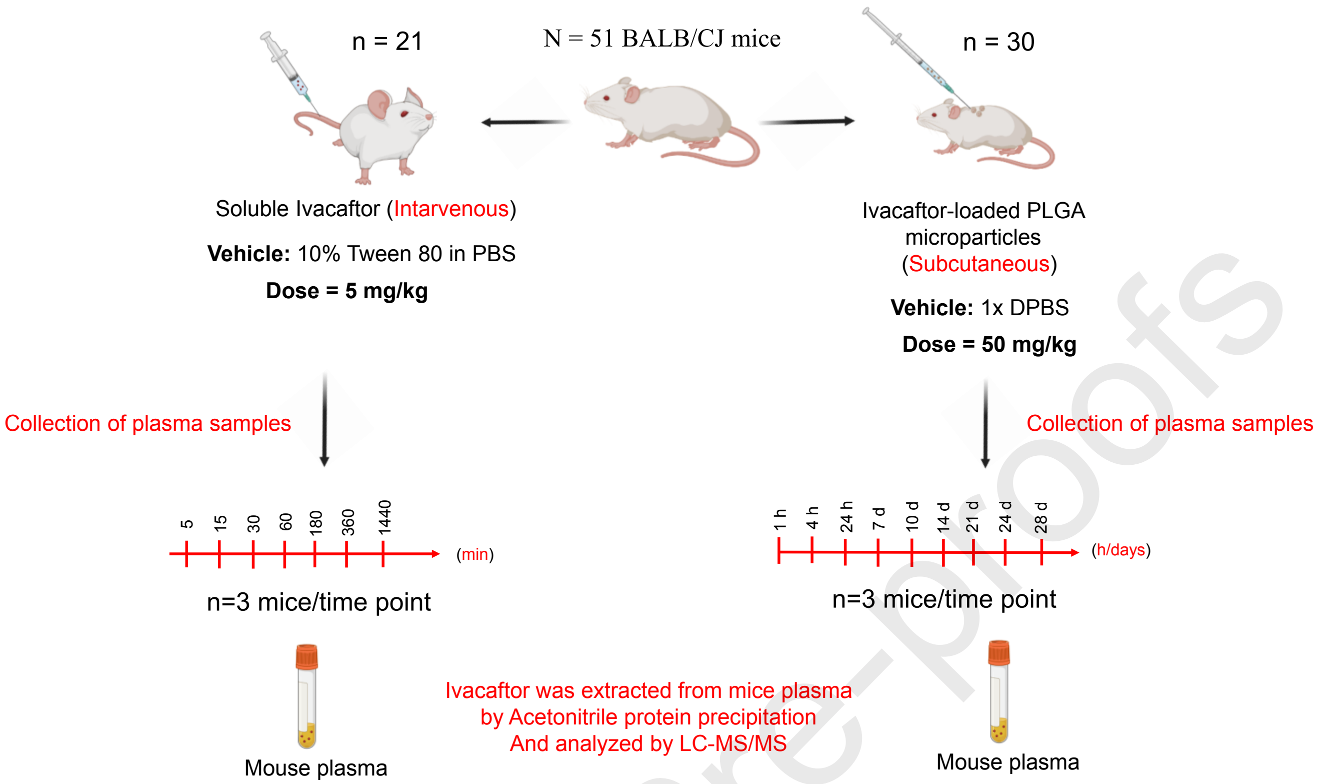

Supplemental Figure 2: A graphical depiction of the experimental design to study the pharmacokinetics of ivacaftor in mice following the SC administration of 50 mg/kg dose of ivacaftor microparticles to a group of 30 mice and the IV administration of a dose of 5 mg/kg ivacaftor (dissolved in 10% Tween 80 in PBS) to a group of 21 mice. Plasma samples were collected at different time points and ivacaftor concentration were determined using LC-MS/MS.

Journal Pre-proofs

Journal Pre-proofs

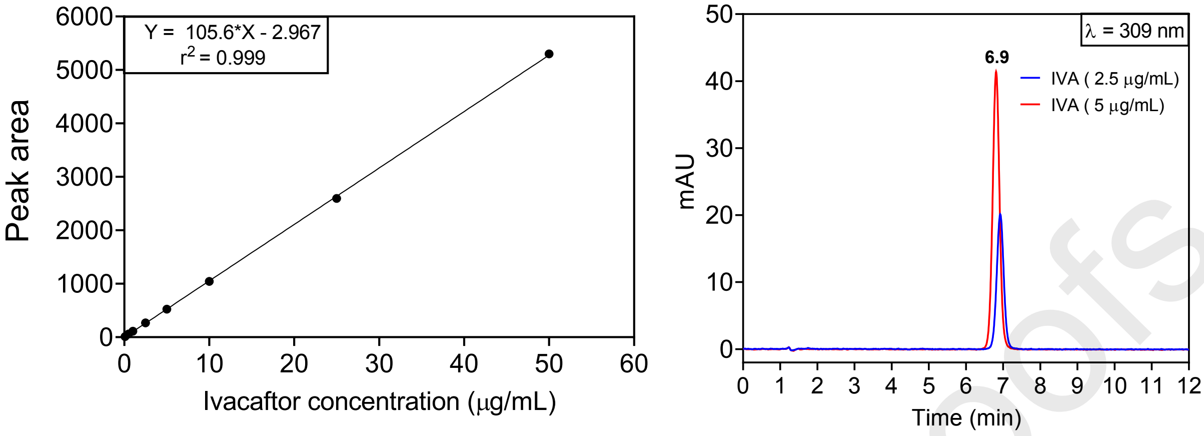

Supplemental Figure 3: Calibration curve (left) and representative chromatogram (right) of ivacaftor in aqueous vehicle. IVA: ivacaftor.

A-

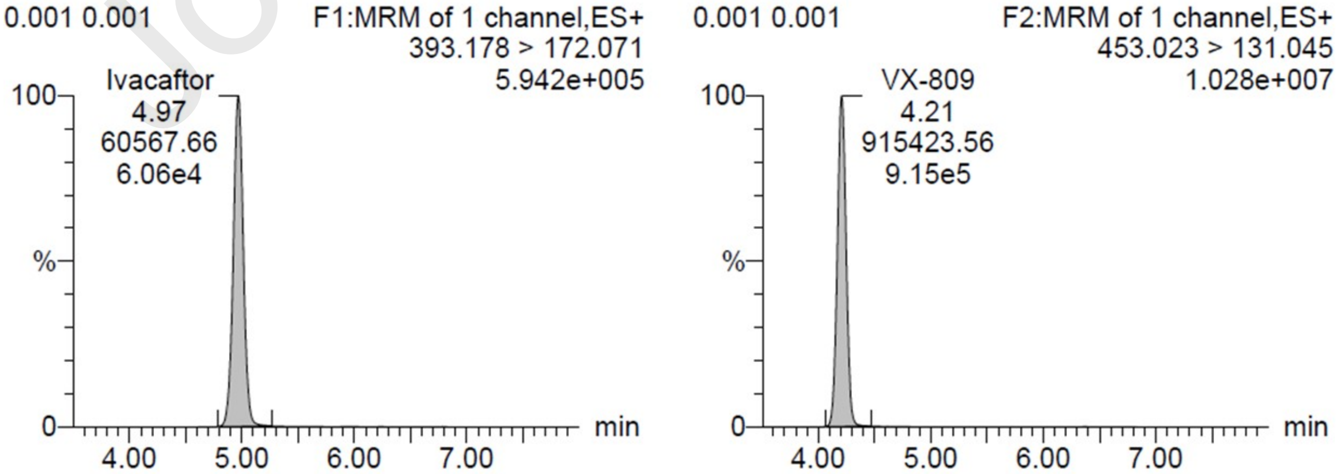

B-

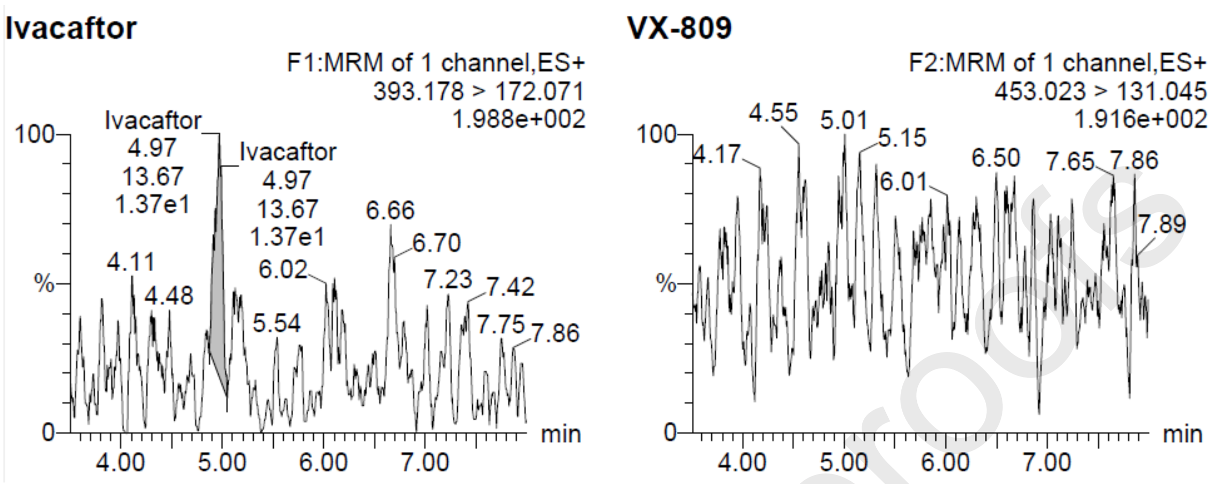

Supplemental Figure 4: A- Representative LC-MS/MS chromatograms for ivacaftor (0.001 µg/mL) and lumacaftor (VX-809) (0.25 µg/mL) following extraction from mice plasma. B- Representative chromatogram of blank plasma sample.

Journal Pre-proofs
